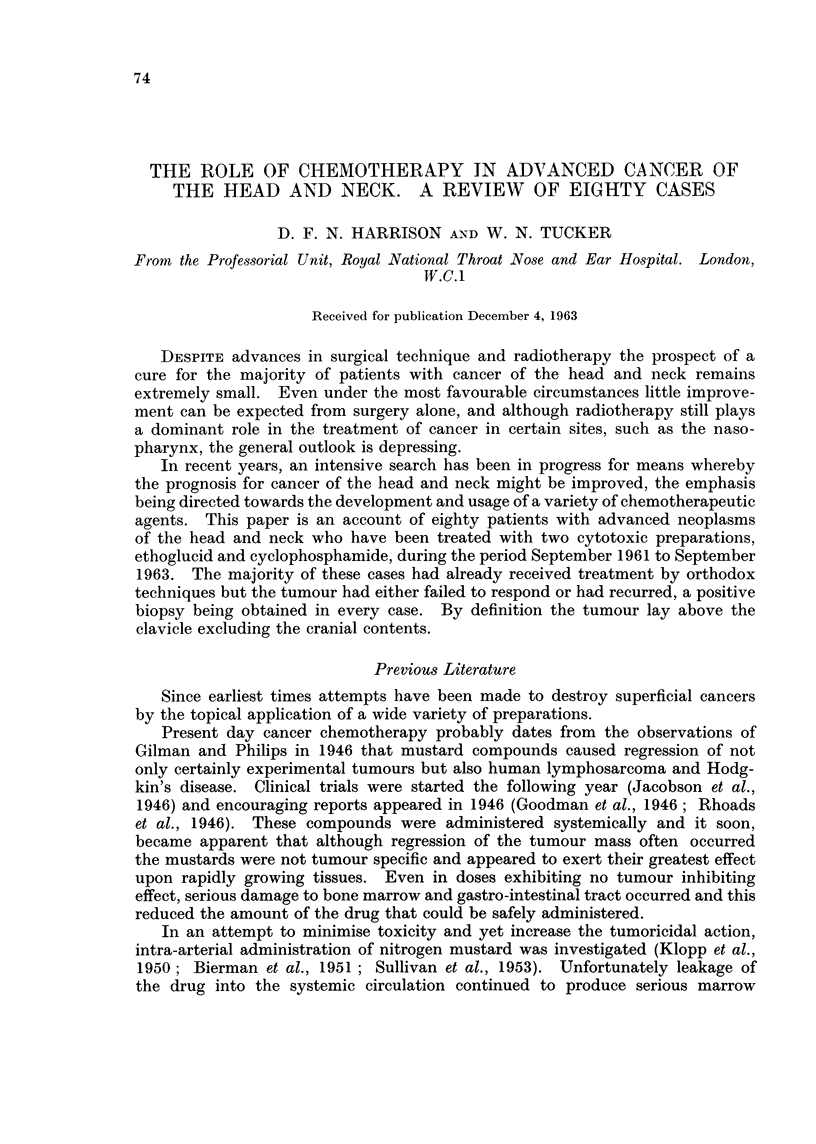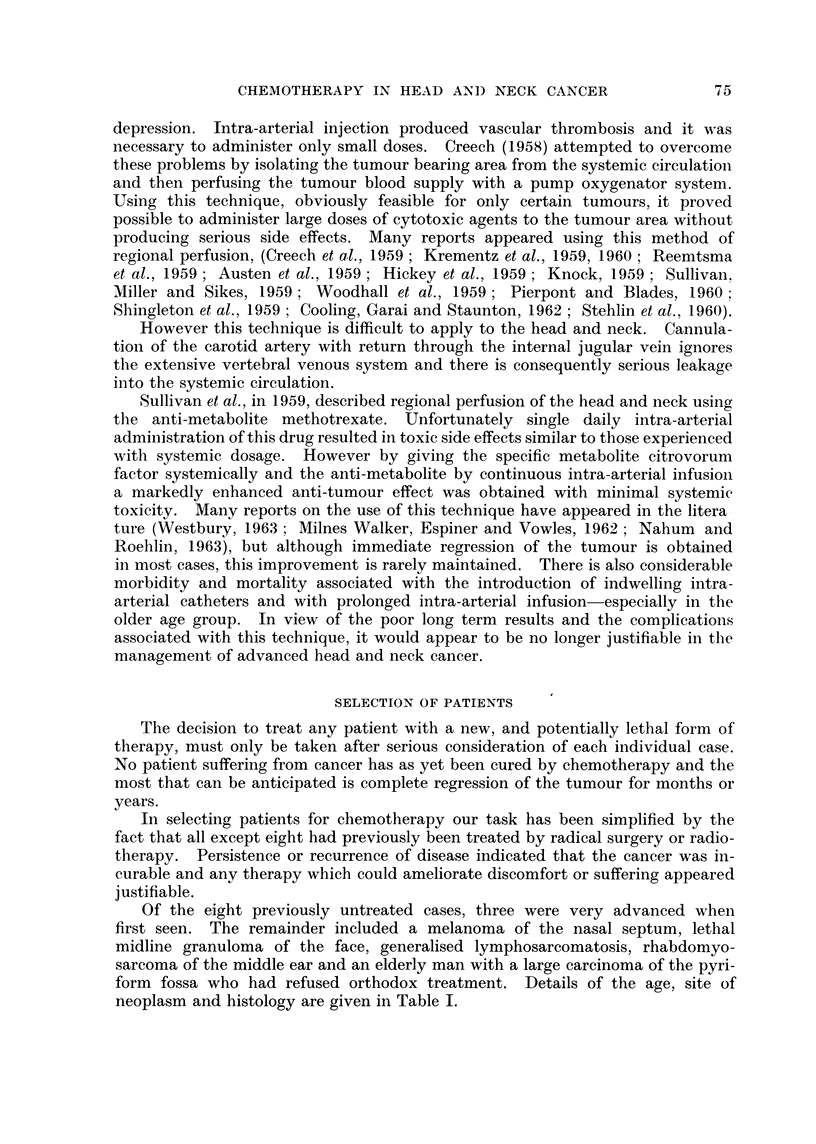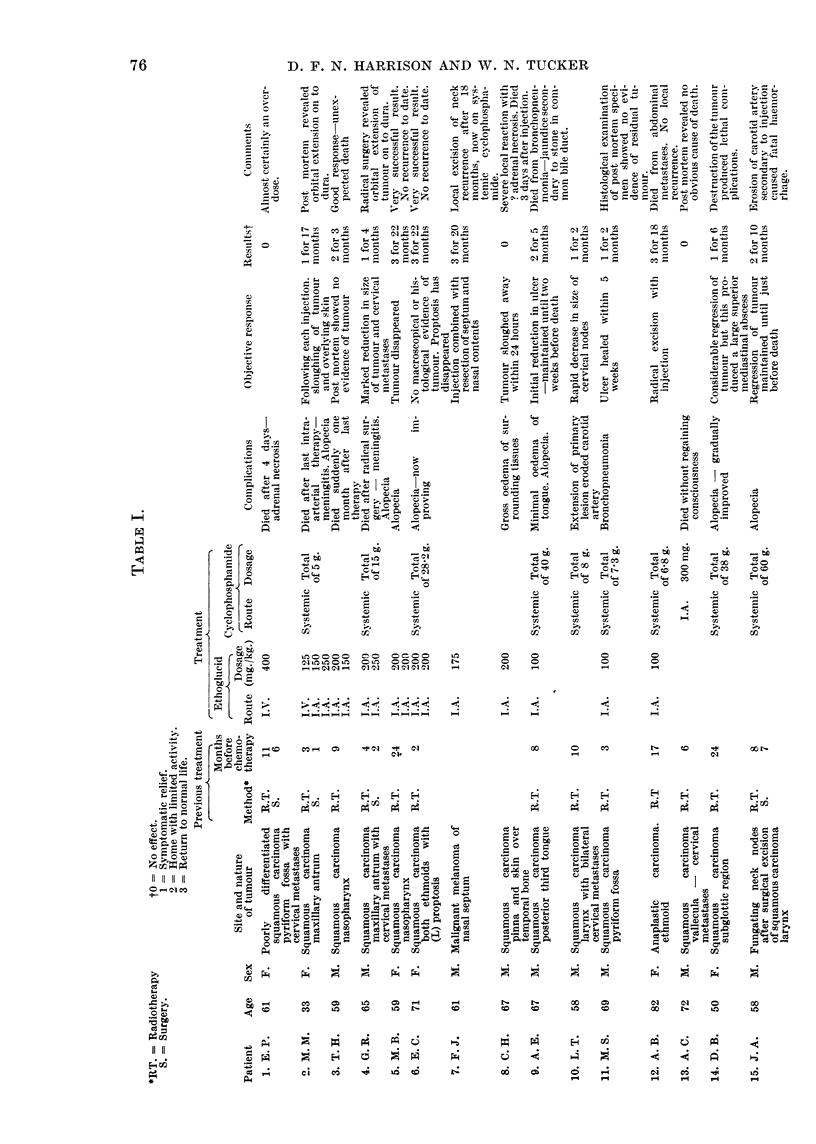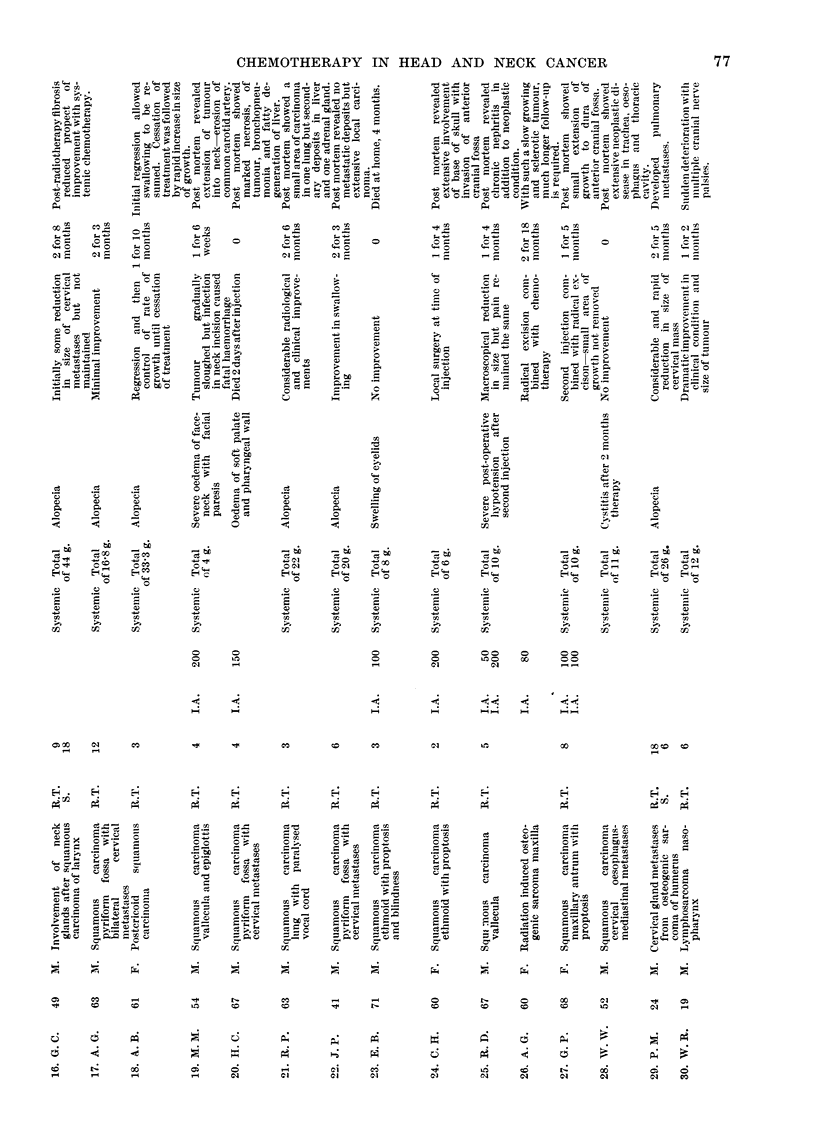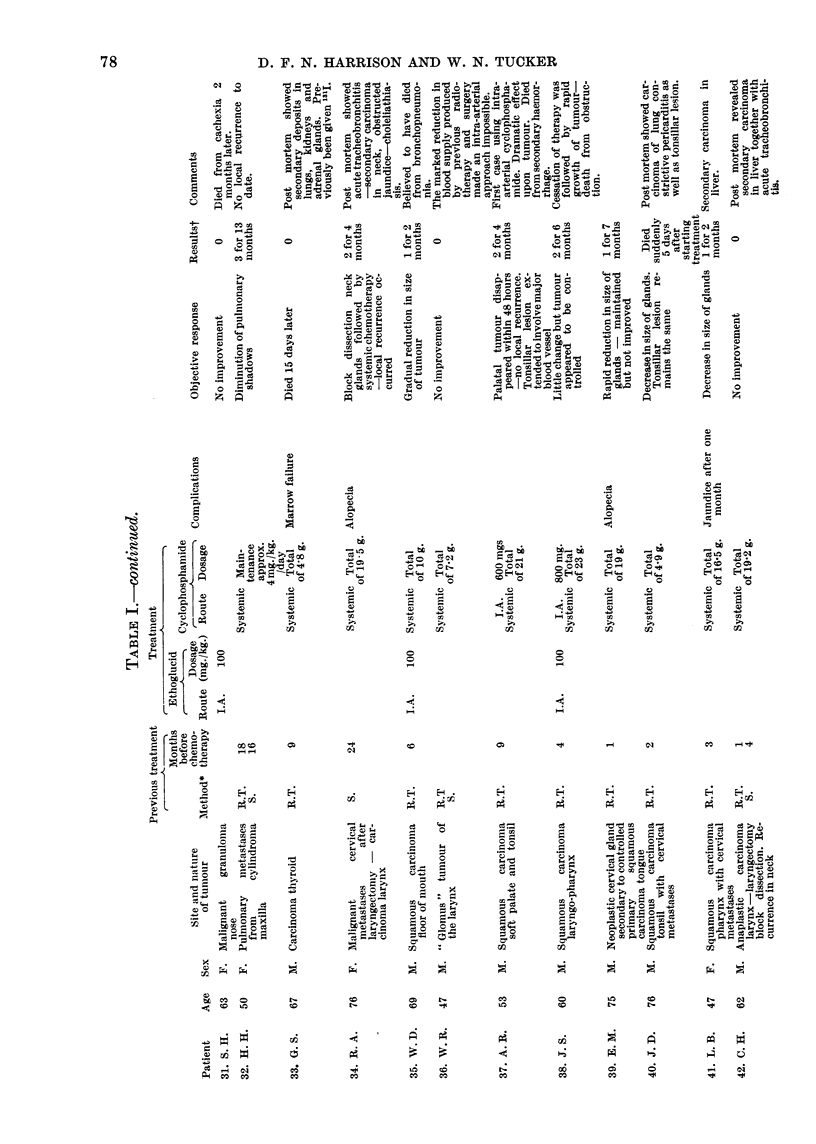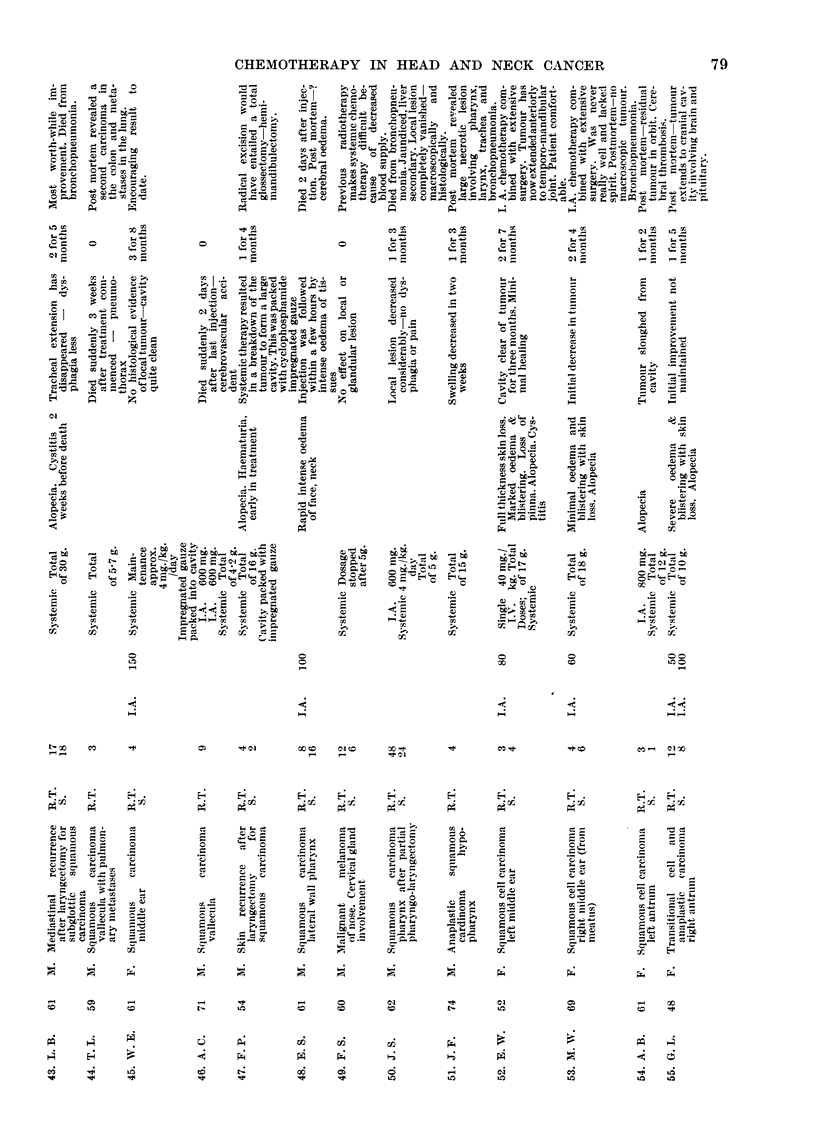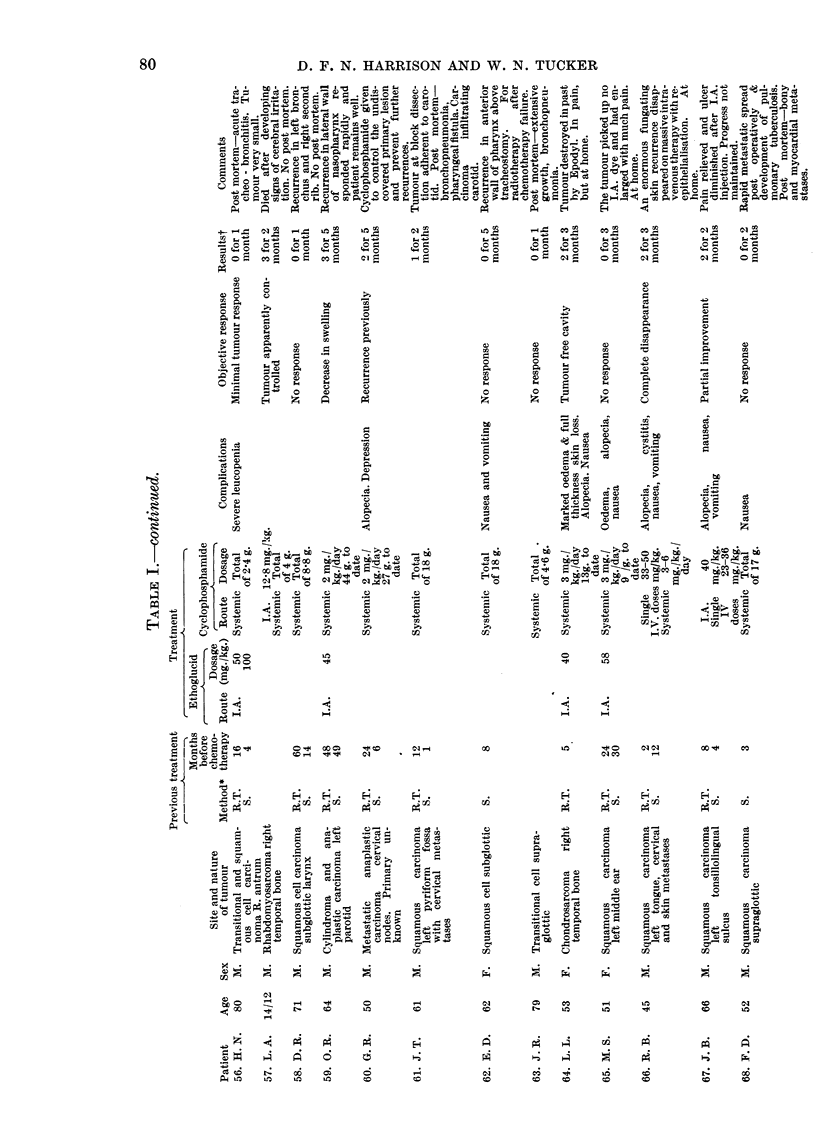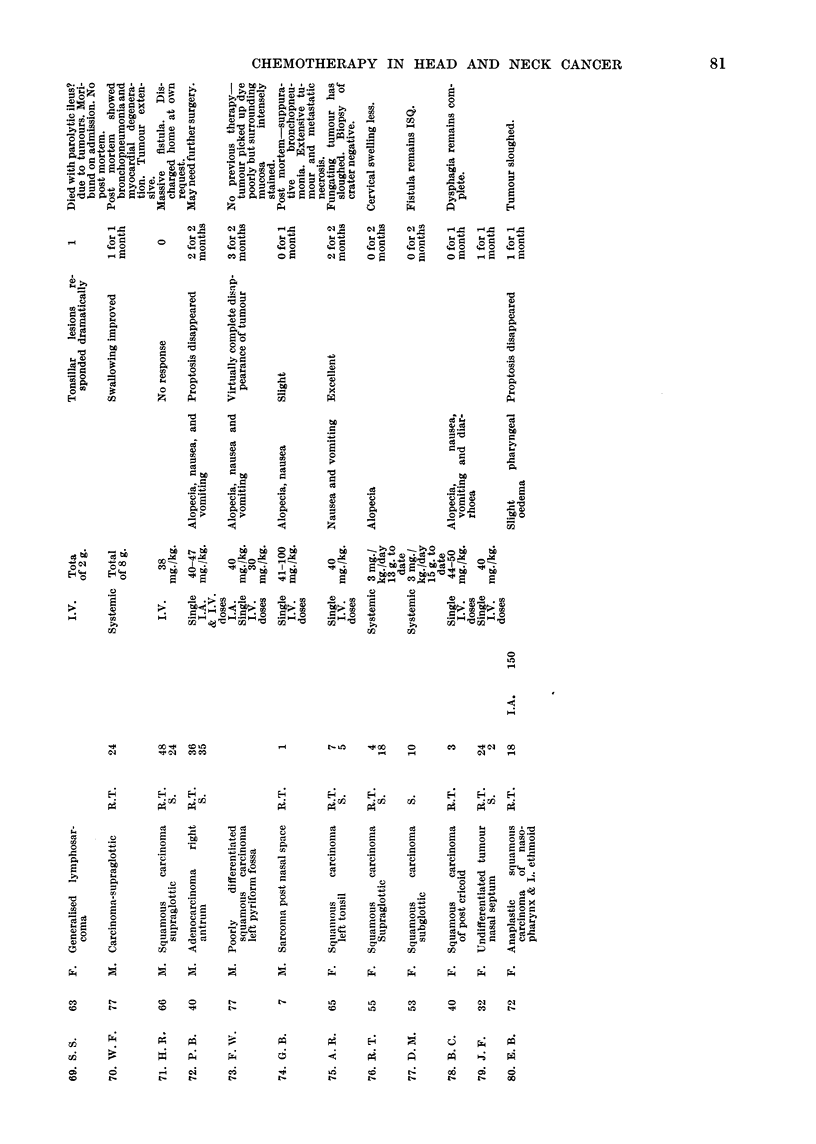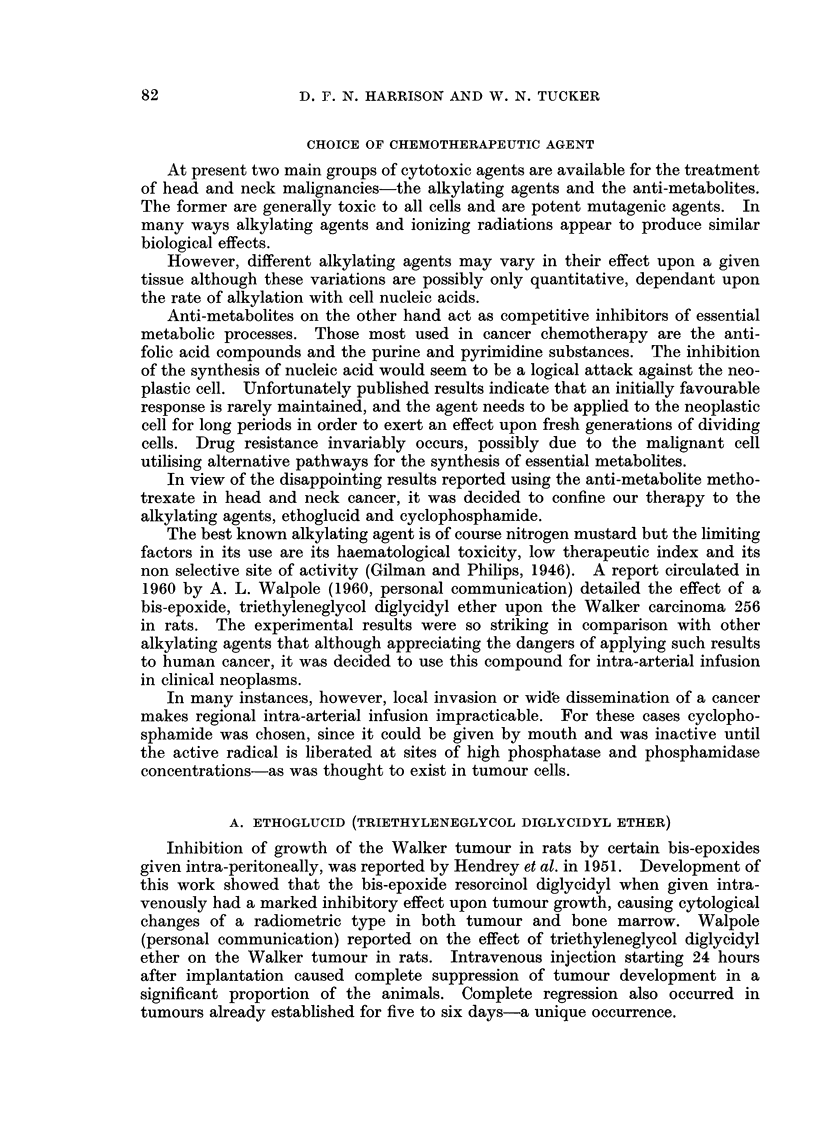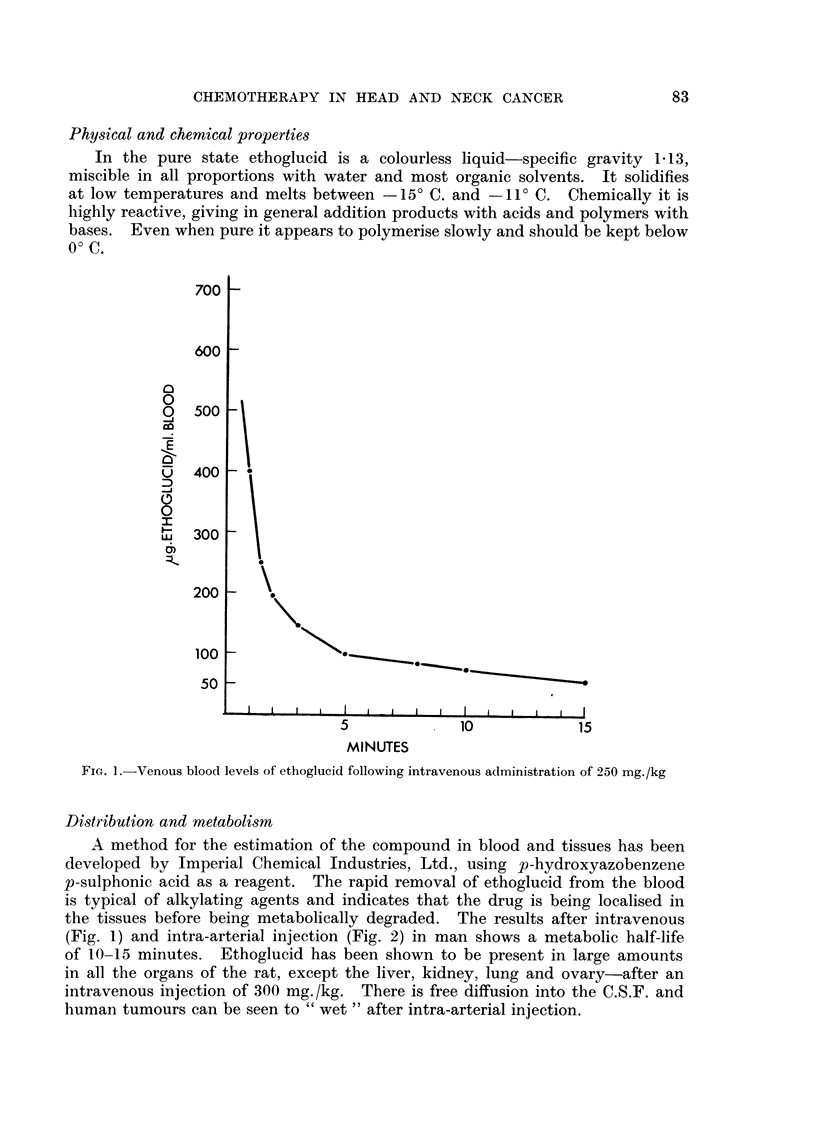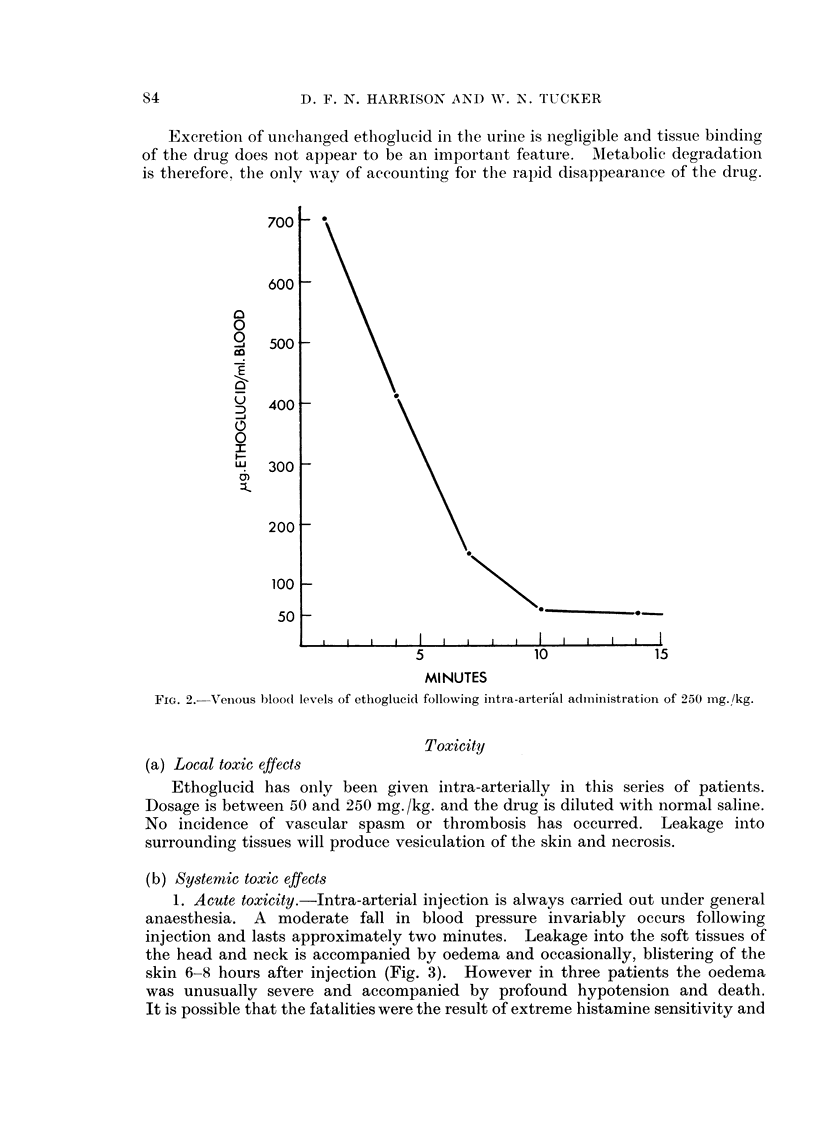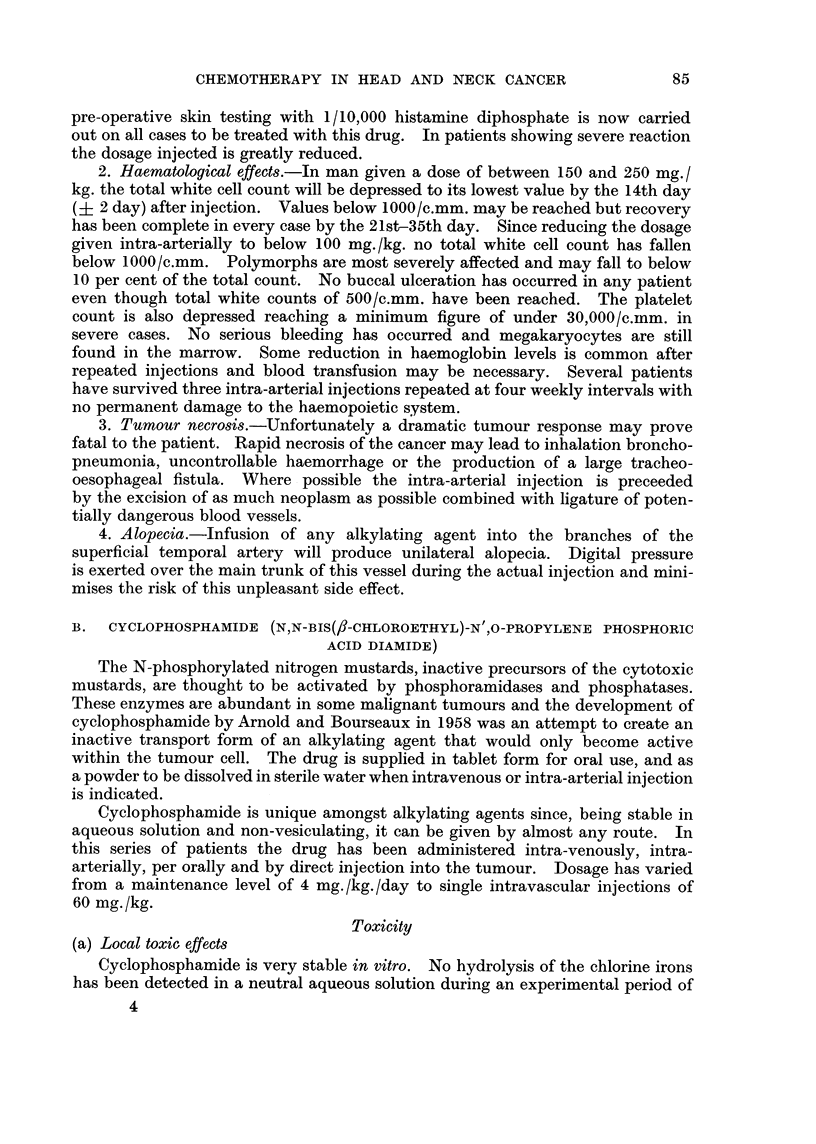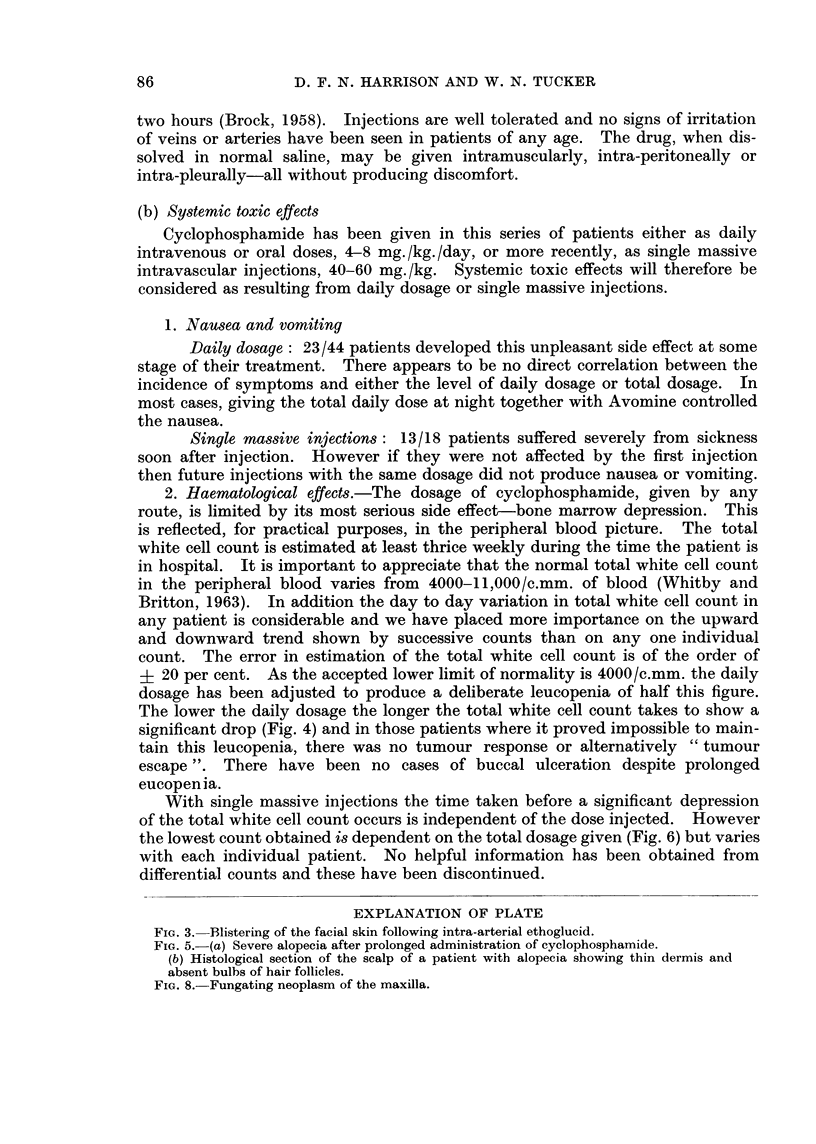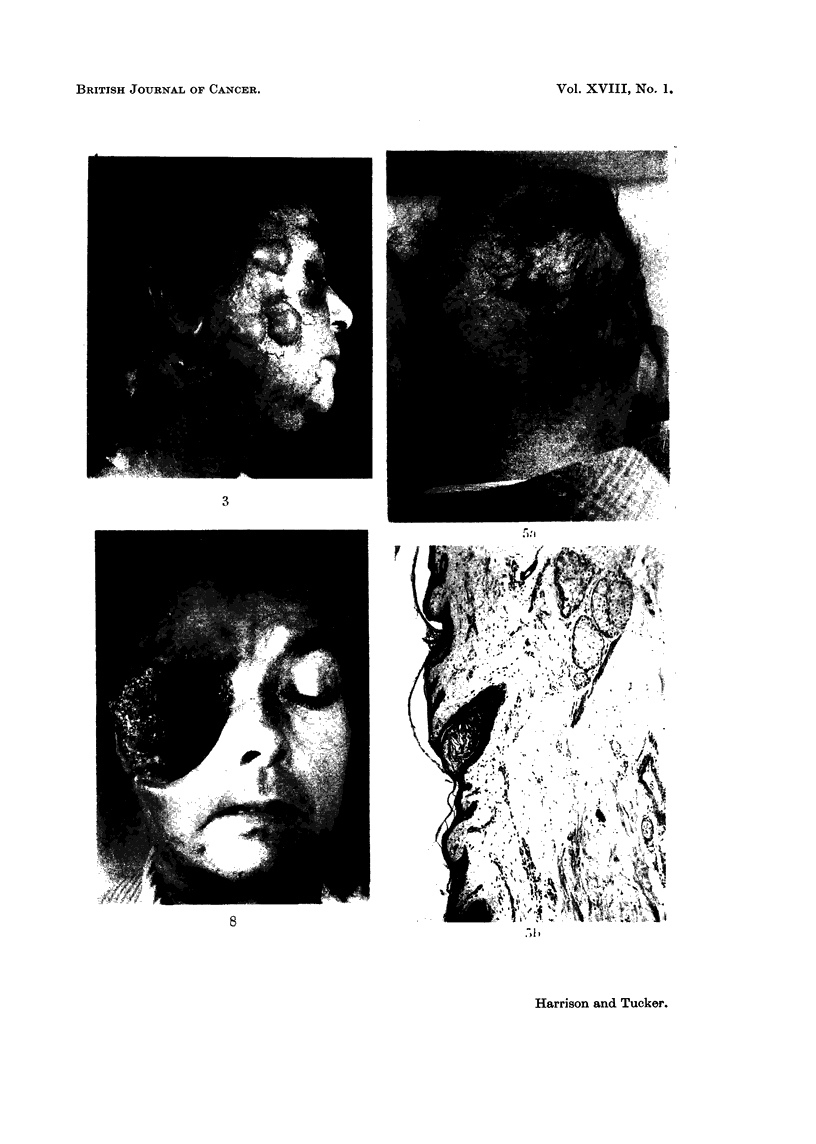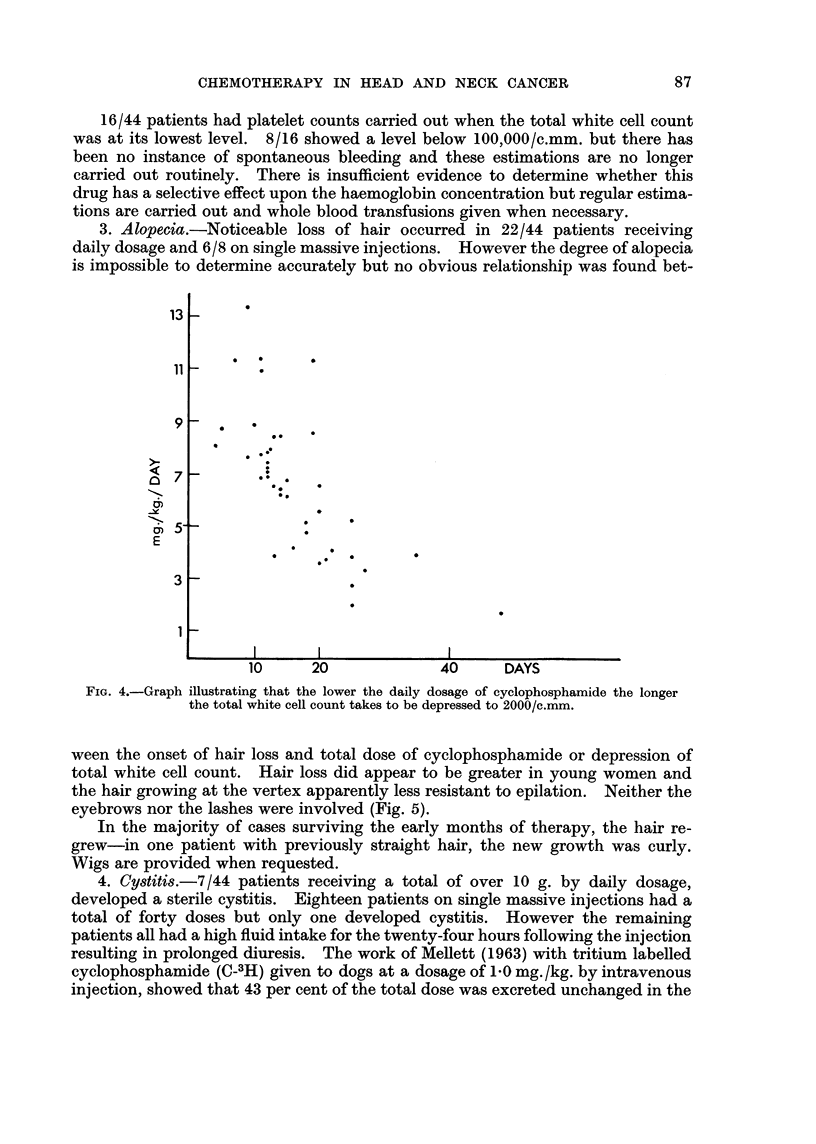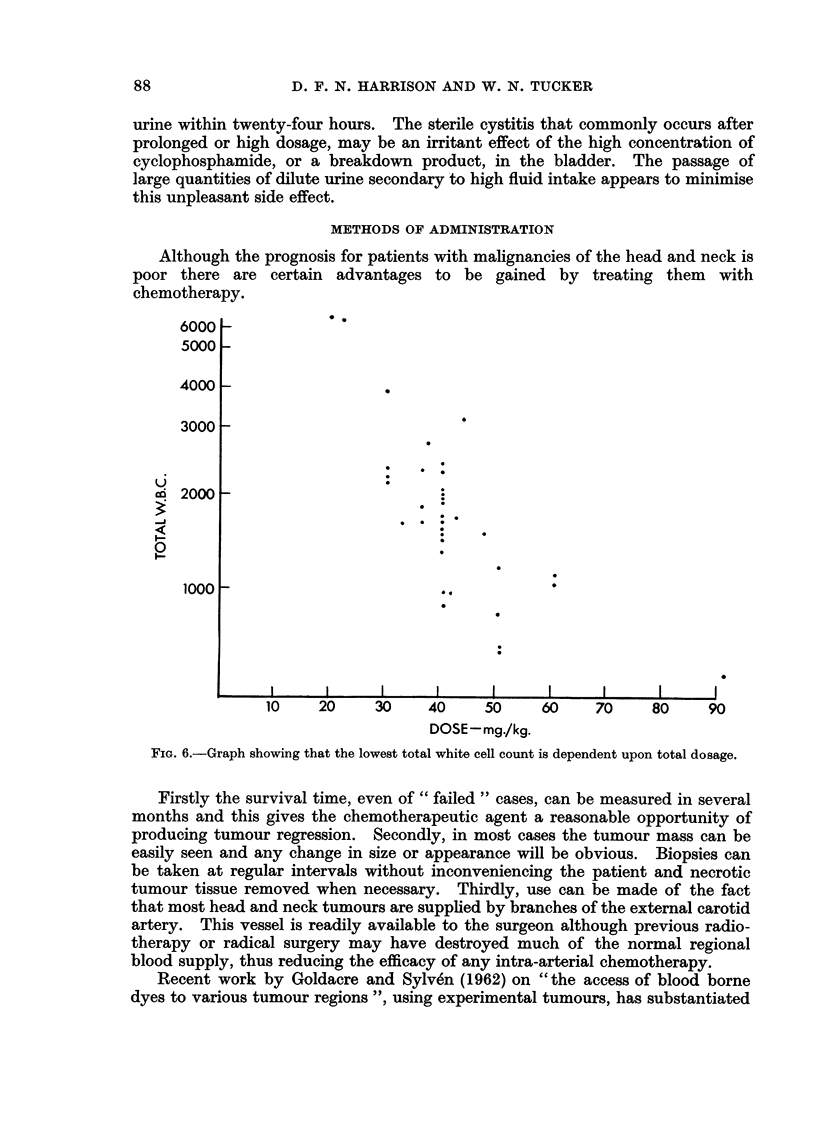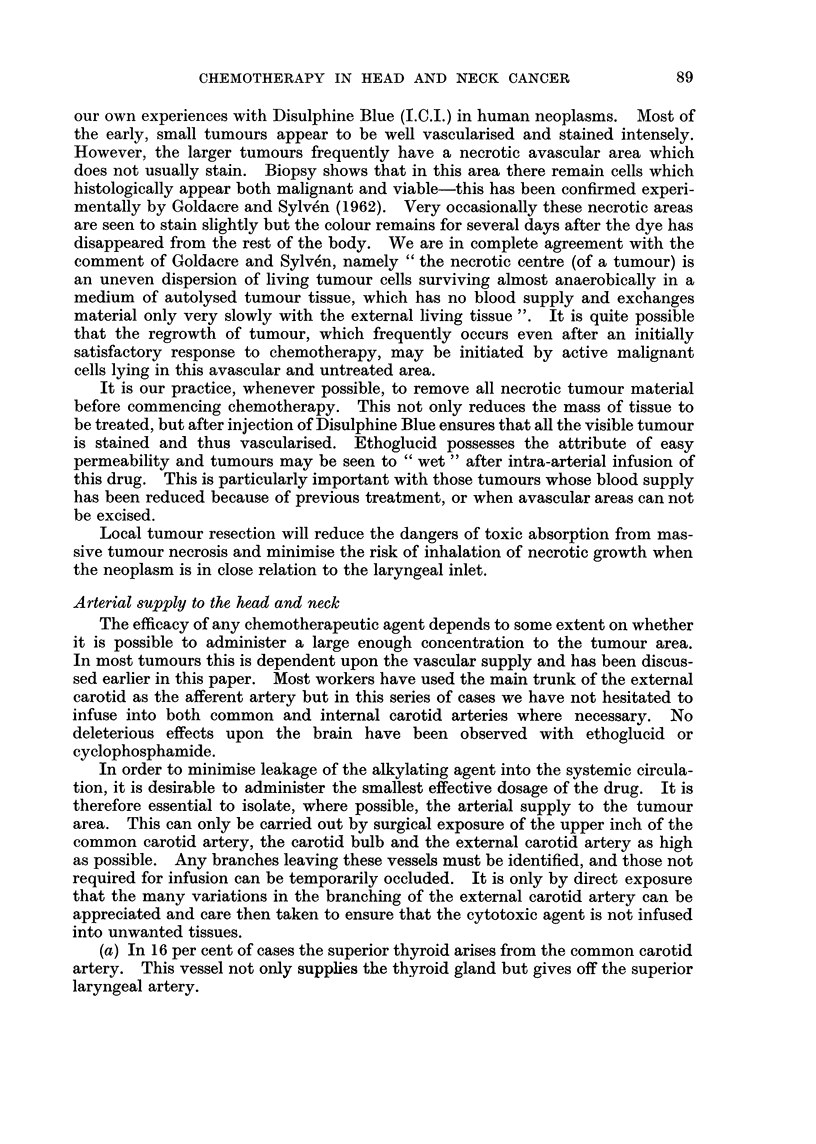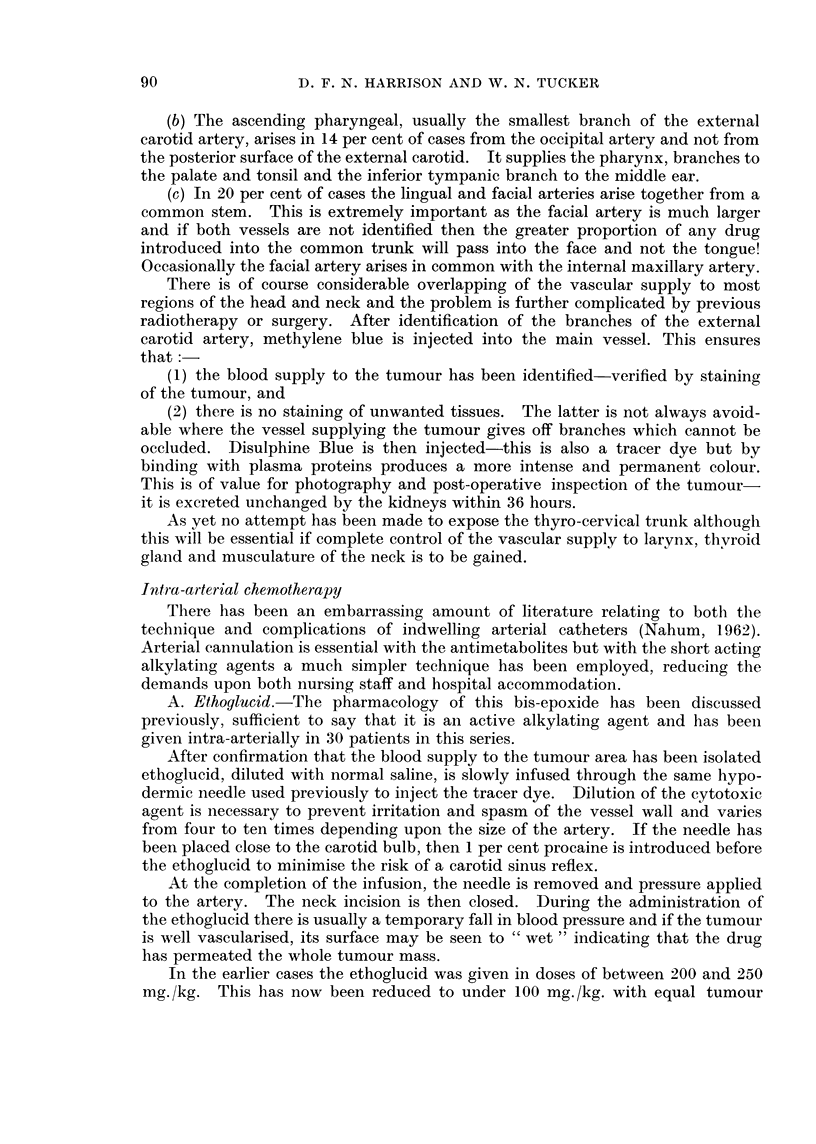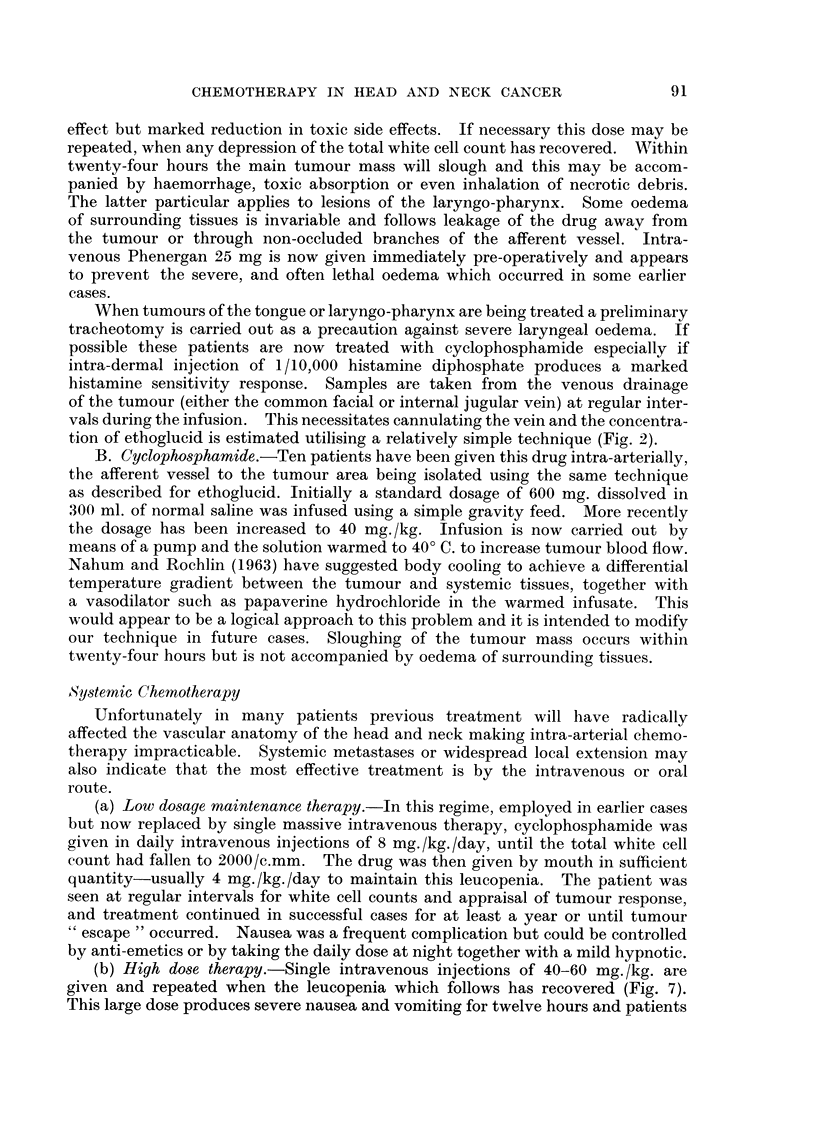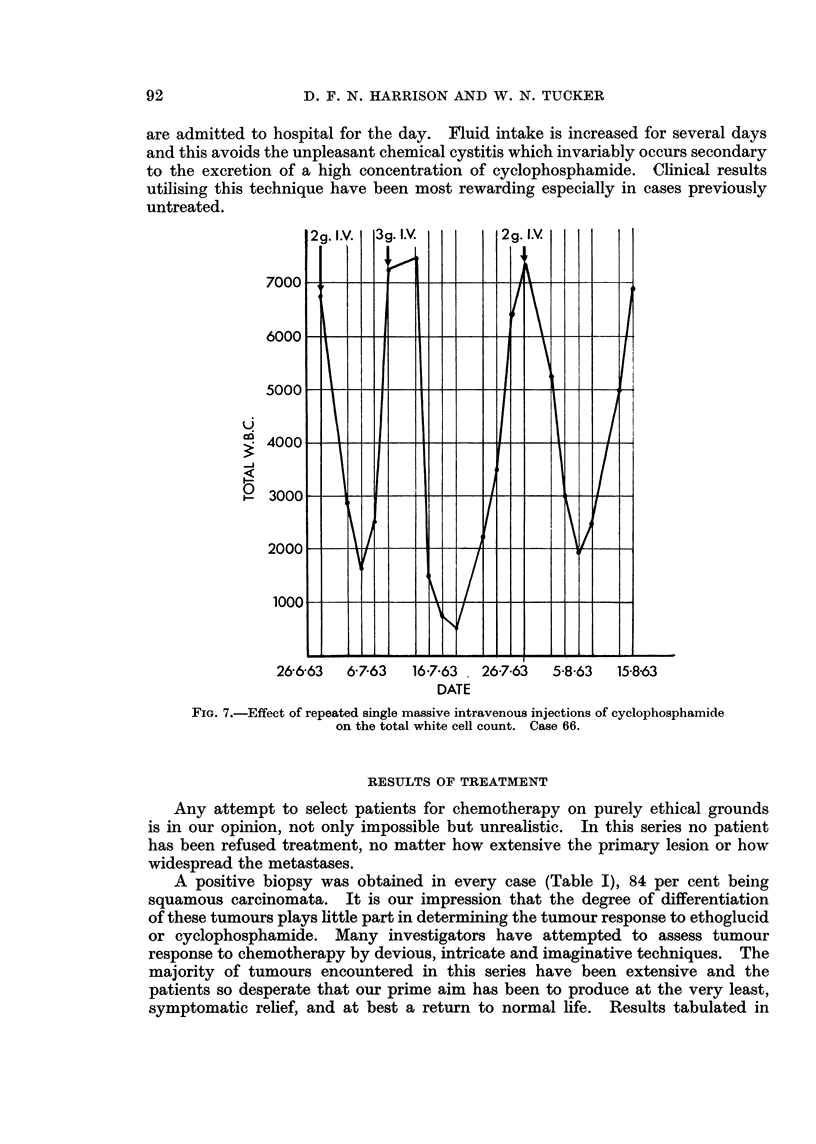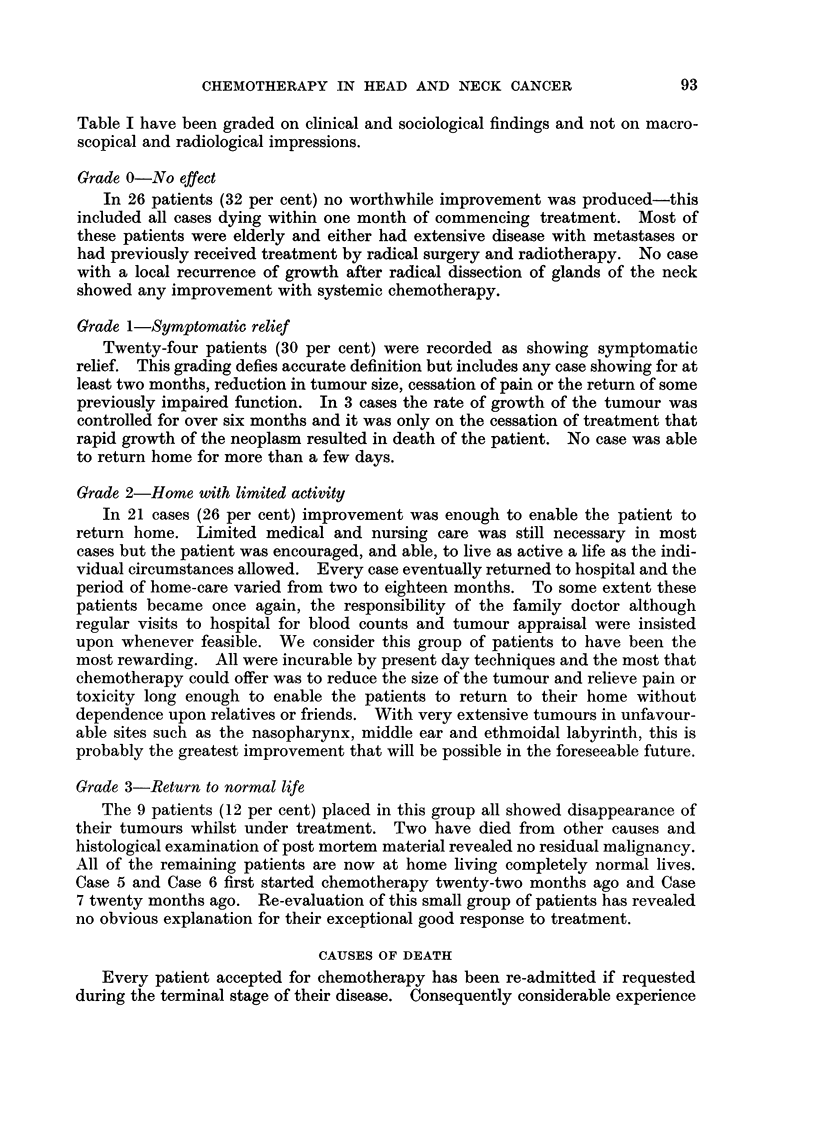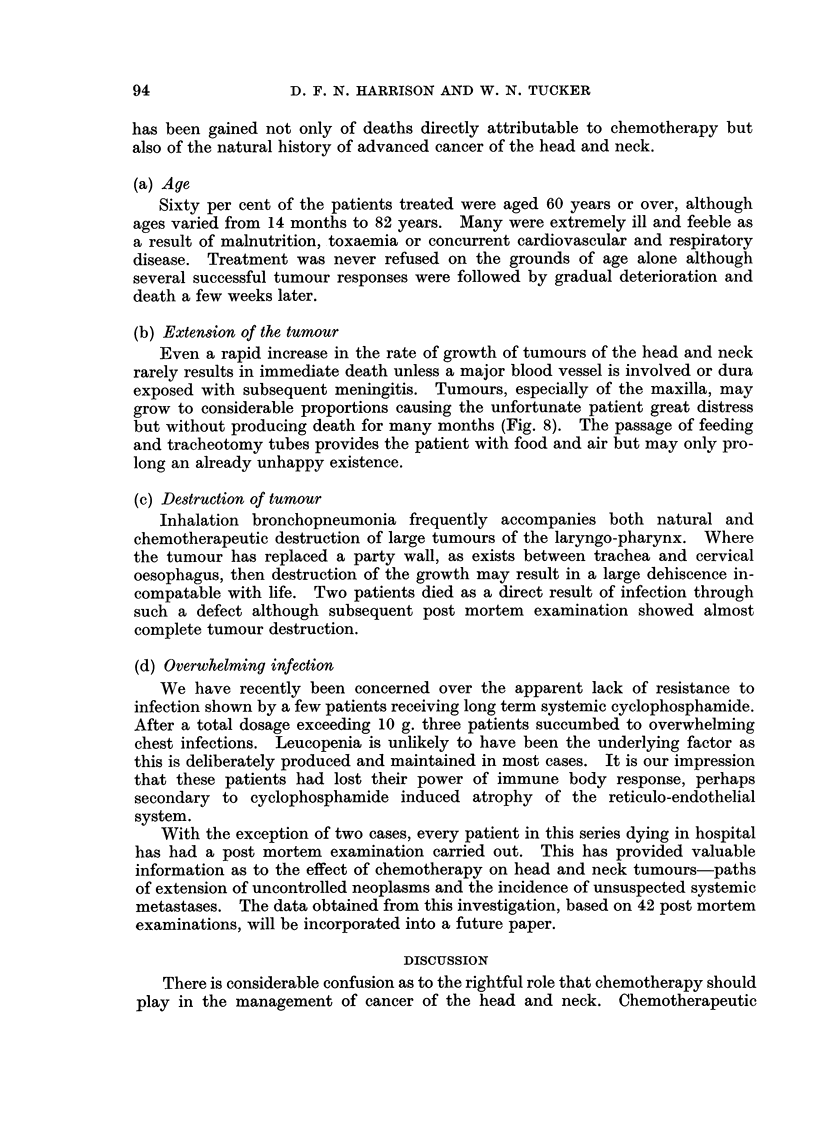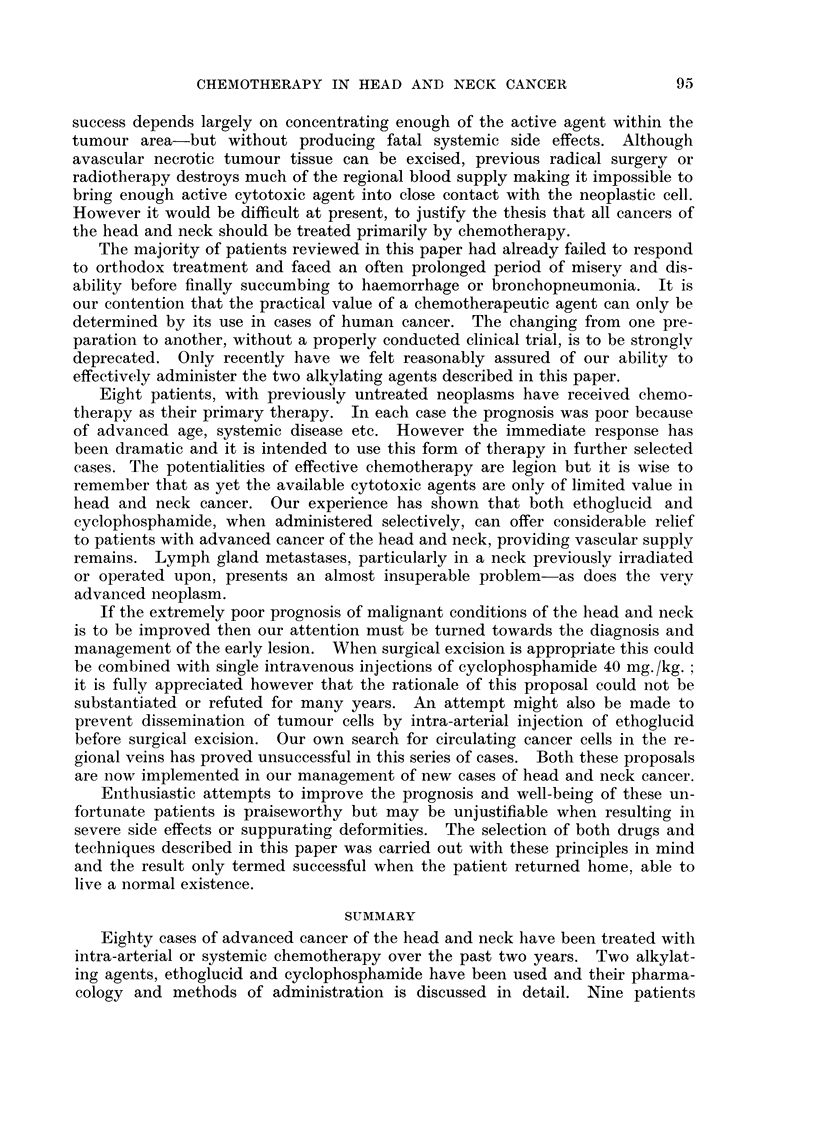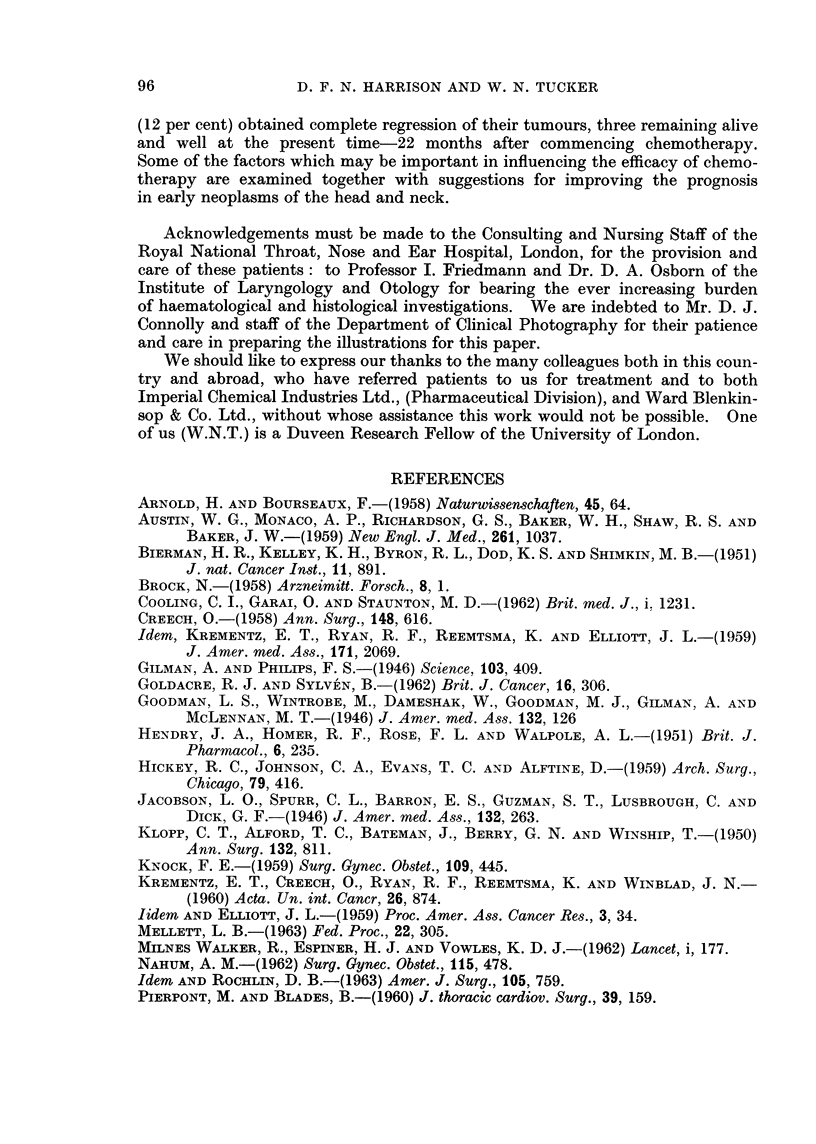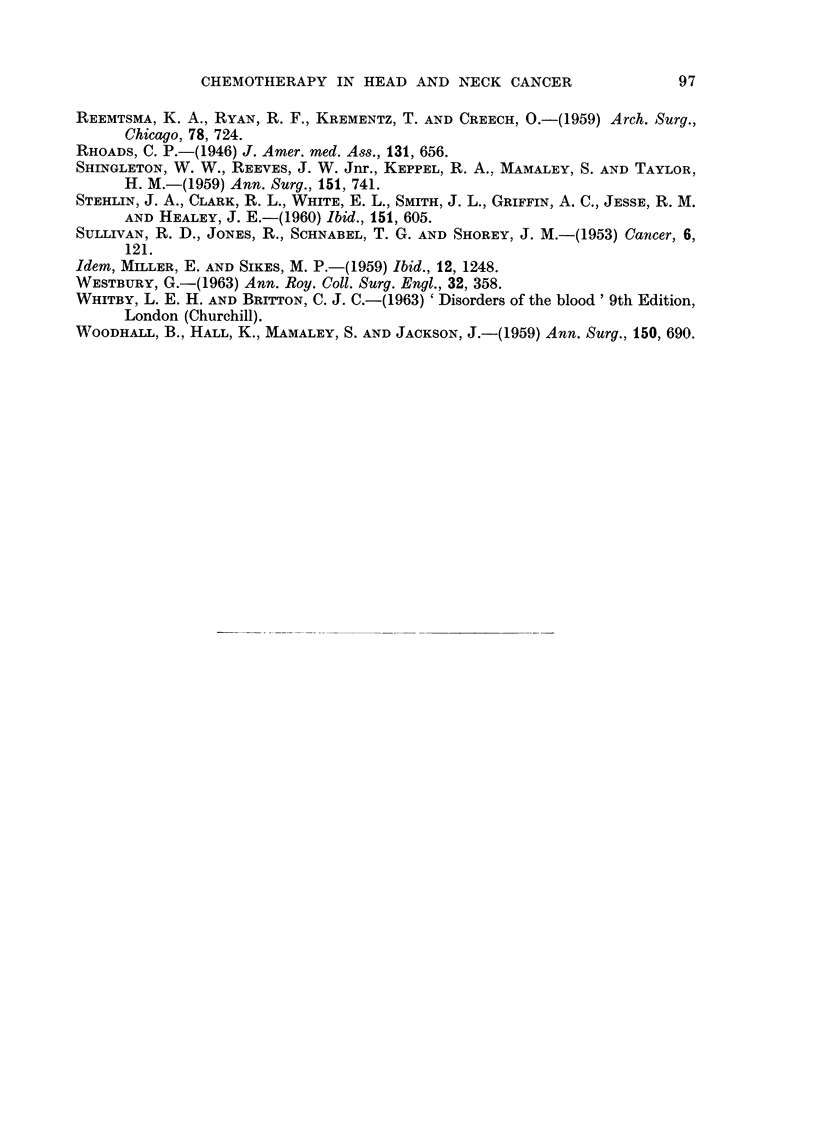# The Role of Chemotherapy in Advanced Cancer of the Head and Neck. A Review of Eighty Cases

**DOI:** 10.1038/bjc.1964.8

**Published:** 1964-03

**Authors:** D. F. N. Harrison, W. N. Tucker

## Abstract

**Images:**


### THE ROLE OF CHEMOTHERAPY IN ADVANCED CANCER OF THE HEAD AND NECK. A REVIEW OF EIGHTY CASES

D. F. N. HARRISON AND W. N. TUCKER

*From the Professorial Unit, Royal National Throat Nose and Ear Hospital. London, W.C.1*

Received for publication December 4, 1963

DESPITE advances in surgical technique and radiotherapy the prospect of a cure for the majority of patients with cancer of the head and neck remains extremely small. Even under the most favourable circumstances little improvement can be expected from surgery alone, and although radiotherapy still plays a dominant role in the treatment of cancer in certain sites, such as the nasopharynx, the general outlook is depressing.

In recent years, an intensive search has been in progress for means whereby the prognosis for cancer of the head and neck might be improved, the emphasis being directed towards the development and usage of a variety of chemotherapeutic agents. This paper is an account of eighty patients with advanced neoplasms of the head and neck who have been treated with two cytotoxic preparations, ethoglucid and cyclophosphamide, during the period September 1961 to September 1963. The majority of these cases had already received treatment by orthodox techniques but the tumour had either failed to respond or had recurred, a positive biopsy being obtained in every case. By definition the tumour lay above the clavicle excluding the cranial contents.

### *Previous Literature*

Since earliest times attempts have been made to destroy superficial cancers by the topical application of a wide variety of preparations.

Present day cancer chemotherapy probably dates from the observations of Gilman and Philips in 1946 that mustard compounds caused regression of not only certainly experimental tumours but also human lymphosarcoma and Hodgkin's disease. Clinical trials were started the following year (Jacobson *et al.*, 1946) and encouraging reports appeared in 1946 (Goodman *et al.*, 1946 ; Rhoads *et al.*, 1946). These compounds were administered systemically and it soon, became apparent that although regression of the tumour mass often occurred the mustards were not tumour specific and appeared to exert their greatest effect upon rapidly growing tissues. Even in doses exhibiting no tumour inhibiting effect, serious damage to bone marrow and gastro-intestinal tract occurred and this reduced the amount of the drug that could be safely administered.

In an attempt to minimise toxicity and yet increase the tumoricidal action, intra-arterial administration of nitrogen mustard was investigated (Klopp *et al.*, 1950 ; Bierman *et al.*, 1951 ; Sullivan *et al.*, 1953). Unfortunately leakage of the drug into the systemic circulation continued to produce serious marrow depression. Intra-arterial injection produced vascular thrombosis and it was necessary to administer only small doses. Creech (1958) attempted to overcome these problems by isolating the tumour bearing area from the systemic circulation and then perfusing the tumour blood supply with a pump oxygenator system. Using this technique, obviously feasible for only certain tumours, it proved possible to administer large doses of cytotoxic agents to the tumour area without producing serious side effects. Many reports appeared using this method of regional perfusion, (Creech *et al.*, 1959 ; Krementz *et al.*, 1959, 1960 ; Reemtsma *et al.*, 1959 ; Austen *et al.*, 1959 ; Hickey *et al.*, 1959 ; Knock, 1959 ; Sullivan, Miller and Sikes, 1959 ; Woodhall *et al.*, 1959 ; Pierpont and Blades, 1960 ; Shingleton *et al.*, 1959 ; Cooling, Garai and Staunton, 1962 ; Stehlin *et al.*, 1960).

However this technique is difficult to apply to the head and neck. Cannulation of the carotid artery with return through the internal jugular vein ignores the extensive vertebral venous system and there is consequently serious leakage into the systemic circulation.

Sullivan *et al.*, in 1959, described regional perfusion of the head and neck using the anti-metabolite methotrexate. Unfortunately single daily intra-arterial administration of this drug resulted in toxic side effects similar to those experienced with systemic dosage. However by giving the specific metabolite citrovorum factor systemically and the anti-metabolite by continuous intra-arterial infusion a markedly enhanced anti-tumour effect was obtained with minimal systemic toxicity. Many reports on the use of this technique have appeared in the literature (Westbury, 1963 ; Milnes Walker, Espiner and Vowles, 1962 ; Nahum and Roehlin, 1963), but although immediate regression of the tumour is obtained in most cases, this improvement is rarely maintained. There is also considerable morbidity and mortality associated with the introduction of indwelling intra-arterial catheters and with prolonged intra-arterial infusion—especially in the older age group. In view of the poor long term results and the complications associated with this technique, it would appear to be no longer justifiable in the management of advanced head and neck cancer.

### SELECTION OF PATIENTS

The decision to treat any patient with a new, and potentially lethal form of therapy, must only be taken after serious consideration of each individual case. No patient suffering from cancer has as yet been cured by chemotherapy and the most that can be anticipated is complete regression of the tumour for months or years.

In selecting patients for chemotherapy our task has been simplified by the fact that all except eight had previously been treated by radical surgery or radiotherapy. Persistence or recurrence of disease indicated that the cancer was incurable and any therapy which could ameliorate discomfort or suffering appeared justifiable.

Of the eight previously untreated cases, three were very advanced when first seen. The remainder included a melanoma of the nasal septum, lethal midline granuloma of the face, generalised lymphosarcomatosis, rhabdomyosarcoma of the middle ear and an elderly man with a large carcinoma of the pyriform fossa who had refused orthodox treatment. Details of the age, site of neoplasm and histology are given in Table I.

### Table I.

*RT. = Radiotherapy

S. = Surgery.

†0 = No effect.

1 = Symptomatic relief.

2 = Home with limited activity.

3 = Return to normal life.

| | | | | Previous treatment | | Treatment | | | | | | | |
|---|---|---|---|---|---|---|---|---|---|---|---|---|---|
| | | | | | | Ethoglucid | | Cyclophosphamide | | | | | |
| Patient | Age | Sex | Site and nature of tumour | Method* | Months before chemo-therapy | Route | Dosage (mg./kg.) | Route | Dosage | Complications | Objective response | Results† | Comments |
| 1. E. P. | 61 | F. | Poorly differentiated squamous carcinoma pyriform fossa with cervical metastases | R.T.<br>S. | 11<br>6 | I.V. | 400 | | | Died after 4 days—adrenal necrosis | | 0 | Almost certainly an overdose. |
| 2. M. M. | 33 | F. | Squamous carcinoma maxillary antrum | R.T.<br>S. | 3<br>1 | I.V.<br>I.A.<br>I.A. | 125<br>150<br>250 | Systemic | Total of 5 g. | Died after last intra-arterial therapy—meningitis. Alopecia | Following each injection, sloughing of tumour and overlying skin | 1 for 17 months | Post mortem revealed orbital extension on to dura. |
| 3. T. H. | 59 | M. | Squamous carcinoma nasopharynx | R.T. | 9 | I.A.<br>I.A. | 200<br>150 | | | Died suddenly one month after last therapy | Post mortem showed no evidence of tumour | 2 for 3 months | Good response—unexpected death |
| 4. G. R. | 65 | M. | Squamous carcinoma maxillary antrum with cervical metastases | R.T.<br>S. | 4<br>2 | I.A.<br>I.A. | 200<br>250 | Systemic | Total of 15 g. | Died after radical surgery — meningitis. Alopecia | Marked reduction in size of tumour and cervical metastases | 1 for 4 months | Radical surgery revealed orbital extension of tumour on to dura. |
| 5. M. B. | 59 | F. | Squamous carcinoma nasopharynx | R.T. | 24 | I.A.<br>I.A. | 200<br>200 | | | Alopecia | Tumour disappeared | 3 for 22 months | Very successful result. No recurrence to date. |
| 6. E. C. | 71 | F. | Squamous carcinoma both ethmoids with (L) proptosis | R.T. | 2 | I.A.<br>I.A. | 200<br>200 | Systemic | Total of 28·2 g. | Alopecia—now improving | No macroscopical or histological evidence of tumour. Proptosis has disappeared | 3 for 22 months | Very successful result. No recurrence to date. |
| 7. F. J. | 61 | M. | Malignant melanoma of nasal septum | | | I.A. | 175 | | | | Injection combined with resection of septum and nasal contents | 3 for 20 months | Local excision of neck recurrence after 18 months, now on systemic cyclophosphamide. |
| 8. C. H. | 67 | M. | Squamous carcinoma pinna and skin over temporal bone | | | I.A. | 200 | | | Gross oedema of surrounding tissues | Tumour sloughed away within 24 hours | 0 | Severe local reaction with ? adrenal necrosis. Died 3 days after injection. |
| 9. A. E. | 67 | M. | Squamous carcinoma posterior third tongue | R.T. | 8 | I.A. | 100 | Systemic | Total of 40 g. | Minimal oedema of tongue. Alopecia. | Initial reduction in ulcer—maintained until two weeks before death | 2 for 5 months | Died from bronchopneumonia—jaundice secondary to stone in common bile duct. |
| 10. L. T. | 58 | M. | Squamous carcinoma larynx with bilateral cervical metastases | R.T. | 10 | | | Systemic | Total of 8 g. | Extension of primary lesion eroded carotid artery | Rapid decrease in size of cervical nodes | 1 for 2 months | |
| 11. M. S. | 69 | M. | Squamous carcinoma pyriform fossa | R.T. | 3 | I.A. | 100 | Systemic | Total of 7·3 g. | Bronchopneumonia | Ulcer healed within 5 weeks | 1 for 2 months | Histological examination of post mortem specimen showed no evidence of residual tumour. |
| 12. A. B. | 82 | F. | Anaplastic carcinoma. ethmoid | R.T | 17 | I.A. | 100 | Systemic | Total of 6·8 g. | | Radical excision with injection | 3 for 18 months | Died from abdominal metastases. No local recurrence. |
| 13. A. C. | 72 | M. | Squamous carcinoma vallecula — cervical metastases | R.T. | 6 | | | I.A. | 300 mg. | Died without regaining consciousness | | 0 | Post mortem revealed no obvious cause of death. |
| 14. D. B. | 50 | F. | Squamous carcinoma subglottic region | R.T. | 24 | | | Systemic | Total of 38 g. | Alopecia — gradually improved | Considerable regression of tumour but this produced a large superior mediastinal abscess | 1 for 6 months | Destruction of the tumour produced lethal complications. |
| 15. J. A. | 58 | M. | Fungating neck nodes after surgical excision of squamous carcinoma larynx | R.T.<br>S. | 8<br>7 | | | Systemic | Total of 60 g. | Alopecia | Regression of tumour maintained until just before death | 2 for 10 months | Erosion of carotid artery secondary to injection caused fatal haemorrhage. |

| | | | | | | | | | | | | |
|---|---|---|---|---|---|---|---|---|---|---|---|---|
| 16. G. C. | 49 | M. | Involvement of neck glands after squamous carcinoma of larynx | R.T.<br>S. | 9<br>18 | | | Systemic Total of 44 g. | Alopecia | Initially some reduction in size of cervical metastases but not maintained | 2 for 8 months | Post-radiotherapy fibrosis reduced propect of improvement with systemic chemotherapy. |
| 17. A. G. | 63 | M. | Squamous carcinoma pyriform fossa with bilateral cervical metastases | R.T. | 12 | | | Systemic Total of 16·8 g. | Alopecia | Minimal improvement | 2 for 3 months | |
| 18. A. B. | 61 | F. | Postcricoid squamous carcinoma | R.T. | 3 | | | Systemic Total of 33·3 g. | Alopecia | Regression and then control of rate of growth until cessation of treatment | 1 for 10 months | Initial regression allowed swallowing to be resumed. Cessation of treatment was followed by rapid increase in size of growth. |
| 19. M. M. | 54 | M. | Squamous carcinoma vallecula and epiglottis | R.T. | 4 | I.A. | 200 | Systemic Total of 4 g. | Severe oedema of face-neck with facial paresis | Tumour gradually sloughed but infection in neck incision caused fatal haemorrhage | 1 for 6 weeks | Post mortem revealed extension of tumour into neck—erosion of common carotid artery. |
| 20. H. C. | 67 | M. | Squamous carcinoma pyriform fossa with cervical metastases | R.T. | 4 | I.A. | 150 | | Oedema of soft palate and pharyngeal wall | Died 2 days after injection | 0 | Post mortem showed marked necrosis, of tumour, bronchopneumonia and fatty degeneration of liver. |
| 21. R. P. | 63 | M. | Squamous carcinoma lung with paralysed vocal cord | R.T. | 3 | | | Systemic Total of 22 g. | Alopecia | Considerable radiological and clinical improvements | 2 for 6 months | Post mortem showed a small area of carcinoma in one lung but secondary deposits in liver and one adrenal gland. |
| 22. J. P. | 41 | M. | Squamous carcinoma pyriform fossa with cervical metastases | R.T. | 6 | | | Systemic Total of 20 g. | Alopecia | Improvement in swallowing | 2 for 3 months | Post mortem revealed no metastatic deposits but extensive local carcinoma. |
| 23. E. B. | 71 | M. | Squamous carcinoma ethmoid with proptosis and blindness | R.T. | 3 | I.A. | 100 | Systemic Total of 8 g. | Swelling of eyelids | No improvement | 0 | Died at home, 4 months. |
| 24. C. H. | 60 | F. | Squamous carcinoma ethmoid with proptosis | R.T. | 2 | I.A. | 200 | Systemic Total of 6 g. | | Local surgery at time of injection | 1 for 4 months | Post mortem revealed extensive involvement of base of skull with invasion of anterior cranial fossa |
| 25. R. D. | 67 | M. | Squamous carcinoma vallecula | R.T. | 5 | I.A.<br>I.A. | 50<br>200 | Systemic Total of 10 g. | Severe post-operative hypotension after second injection | Macroscopical reduction in size but pain remained the same | 1 for 4 months | Post mortem revealed chronic nephritis in addition to neoplastic condition. |
| 26. A. G. | 60 | F. | Radiation induced osteogenic sarcoma maxilla | | | I.A. | 80 | | | Radical excision combined with chemotherapy | 2 for 18 months | With such a slow growing and sclerotic tumour, much longer follow-up is required. |
| 27. G. P. | 68 | F. | Squamous carcinoma maxillary antrum with proptosis | R.T. | 8 | I.A.<br>I.A. | 100<br>100 | Systemic Total of 10 g. | | Second injection combined with radical excison—small area of growth not removed | 1 for 5 months | Post mortem showed small extension of growth to dura of anterior cranial fossa. |
| 28. W. W. | 52 | M. | Squamous carcinoma cervical oesophagus-mediastinal metastases | | | | | Systemic Total of 11 g. | Cystitis after 2 months therapy | No improvement | 0 | Post mortem showed extensive neoplastic disease in trachea, oesophagus and thoracic cavity. |
| 29. P. M. | 24 | M. | Cervical gland metastases from osteogenic sarcoma of humerus | R.T.<br>S. | 18<br>6 | | | Systemic Total of 26 g. | Alopecia | Considerable and rapid reduction in size of cervical mass | 2 for 5 months | Developed pulmonary metastases. |
| 30. W. R. | 19 | M. | Lymphosarcoma nasopharynx | R.T. | 6 | | | Systemic Total of 12 g. | | Dramatic improvement in clinical condition and size of tumour | 1 for 2 months | Sudden deterioration with multiple cranial nerve palsies. |

TABLE I.—*continued.*

| Patient | Age | Sex | Site and nature of tumour | Previous treatment: Method* | Previous treatment: Months before chemotherapy | Treatment: Ethoglucid: Route | Treatment: Ethoglucid: Dosage (mg./kg.) | Treatment: Cyclophosphamide: Route | Treatment: Cyclophosphamide: Dosage | Complications | Objective response | Results† | Comments |
|---|---|---|---|---|---|---|---|---|---|---|---|---|---|
| 31. S. H. | 63 | F. | Malignant granuloma nose | | | I.A. | 100 | | | | No improvement | 0 | Died from cachexia 2 months later. |
| 32. H. H. | 50 | F. | Pulmonary metastases from cylindroma maxilla | R.T.<br>S. | 18<br>16 | | | Systemic | Maintenance approx. 4 mg./kg. /day | | Diminution of pulmonary shadows | 3 for 13 months | No local recurrence to date. |
| 33. G. S. | 67 | M. | Carcinoma thyroid | R.T. | 9 | | | Systemic | Total of 4·8 g. | Marrow failure | Died 15 days later | 0 | Post mortem showed secondary deposits in lungs, kidneys and adrenal glands. Previously been given $^{131}$I. |
| 34. R. A. | 76 | F. | Malignant cervical metastases after laryngectomy — carcinoma larynx | S. | 24 | | | Systemic | Total of 19·5 g. | Alopecia | Block dissection neck glands followed by systemic chemotherapy —local recurrence occurred | 2 for 4 months | Post mortem showed acute tracheobronchitis —secondary carcinoma in neck, obstructed jaundice—choleliathiasis. |
| 35. W. D. | 69 | M. | Squamous carcinoma floor of mouth | R.T. | 6 | I.A. | 100 | Systemic | Total of 10 g. | | Gradual reduction in size of tumour | 1 for 2 months | Believed to have died from bronchopneumonia. |
| 36. W. R. | 47 | M. | "Glomus" tumour of the larynx | R.T<br>S. | | | | Systemic | Total of 7·2 g. | | No improvement | 0 | The marked reduction in blood supply produced by previous radiotherapy and surgery made an intra-arterial approach impossible. |
| 37. A. R. | 53 | M. | Squamous carcinoma soft palate and tonsil | R.T. | 9 | | | I.A.<br>Systemic | 600 mgs<br>Total of 21 g. | | Palatal tumour disappeared within 48 hours —no local recurrence. Tonsillar lesion extended to involve major blood vessel | 2 for 4 months | First case using intra-arterial cyclophosphamide. Dramatic effect upon tumour. Died from secondary haemorrhage. |
| 38. J. S. | 60 | M. | Squamous carcinoma laryngo-pharynx | R.T. | 4 | I.A. | 100 | I.A.<br>Systemic | 800 mg.<br>Total of 23 g. | | Little change but tumour appeared to be controlled | 2 for 6 months | Cessation of therapy was followed by rapid growth of tumour—death from obstruction. |
| 39. E. M. | 75 | M. | Neoplastic cervical gland secondary to controlled primary squamous carcinoma tongue | R.T. | 1 | | | Systemic | Total of 19 g. | Alopecia | Rapid reduction in size of glands — maintained but not improved | 1 for 7 months | |
| 40. J. D. | 76 | M. | Squamous carcinoma tonsil with cervical metastases | R.T. | 2 | | | Systemic | Total of 4·9 g. | | Decrease in size of glands. Tonsillar lesion remains the same | Died suddenly 5 days after starting treatment | Post mortem showed carcinoma of lung constrictive pericarditis as well as tonsillar lesion. |
| 41. L. B. | 47 | F. | Squamous carcinoma pharynx with cervical metastases | R.T. | 3 | | | Systemic | Total of 16·5 g. | Jaundice after one month | Decrease in size of glands | 1 for 2 months | Secondary carcinoma in liver. |
| 42. C. H. | 62 | M. | Anaplastic carcinoma larynx—laryngectomy block dissection. Recurrence in neck | R.T.<br>S. | 1<br>4 | | | Systemic | Total of 19·2 g. | | No improvement | 0 | Post mortem revealed secondary carcinoma in liver together with acute tracheobronchitis. |

| | | | | | | | | | | | | |
|---|---|---|---|---|---|---|---|---|---|---|---|---|
| 43. | L. B. | 61 | M. | Mediastinal recurrence after laryngectomy for subglottic squamous carcinoma | R.T.<br>S. | 17<br>18 | | | Systemic Total of 30 g. | Alopecia. Cystitis 2 weeks before death | Tracheal extension has disappeared — dysphagia less | 2 for 5 months | Most worth-while improvement. Died from bronchopneumonia. |
| 44. | T. L. | 59 | M. | Squamous carcinoma vallecula with pulmonary metastases | R.T. | 3 | | | Systemic Total of 5·7 g. | | Died suddenly 3 weeks after treatment commenced — pneumothorax | 0 | Post mortem revealed a second carcinoma in the colon and metastases in the lung. |
| 45. | W. E. | 61 | F. | Squamous carcinoma middle ear | R.T.<br>S. | 4 | I.A. | 150 | Systemic Maintenance approx. 4 mg./kg. /day<br>Impregnated gauze packed into cavity | | No histological evidence of local tumour—cavity quite clean | 3 for 8 months | Encouraging result to date. |
| 46. | A. C. | 71 | M. | Squamous carcinoma vallecula | R.T. | 9 | | | I.A. 600 mg.<br>I.A. 600 mg.<br>Systemic Total of 4·2 g. | | Died suddenly 2 days after last injection—cerebrovascular accident | 0 | |
| 47. | F. P. | 54 | M. | Skin recurrence after laryngectomy for squamous carcinoma | R.T.<br>S. | 4<br>2 | | | Systemic Total of 16 g.<br>Cavity packed with impregnated gauze | Alopecia. Haematuria, early in treatment | Systemic therapy resulted in a breakdown of the tumour to form a large cavity. This was packed with cyclophosphamide impregnated gauze | 1 for 4 months | Radical excision would have entailed a total glossectomy—hemi-mandibulectomy. |
| 48. | E. S. | 61 | M. | Squamous carcinoma lateral wall pharynx | R.T.<br>S. | 8<br>16 | I.A. | 100 | | Rapid intense oedema of face, neck | Injection was followed within a few hours by intense oedema of tissues | | Died 2 days after injection. Post mortem—? cerebral oedema. |
| 49. | F. S. | 60 | M. | Malignant melanoma of nose. Cervical gland involvement | R.T.<br>S. | 12<br>6 | | | Systemic Dosage stopped after 5g. | | No effect on local or glandular lesion | 0 | Previous radiotherapy makes systemic chemotherapy difficult because of decreased blood supply. |
| 50. | J. S. | 62 | M. | Squamous carcinoma pharynx after partial pharyngo-laryngectomy | R.T.<br>S. | 48<br>24 | | | I.A. 600 mg.<br>Systemic 4 mg./kg. day Total of 5 g. | | Local lesion decreased considerably—no dysphagia or pain | 1 for 3 months | Died from bronchopneumonia. Jaundiced, liver secondary. Local lesion completely vanished—macroscopically and histologically. |
| 51. | J. F. | 74 | M. | Anaplastic squamous cardinoma hypopharynx | R.T. | 4 | | | Systemic Total of 15 g. | | Swelling decreased in two weeks | 1 for 3 months | Post mortem revealed large necrotic lesion involving pharynx, larynx, trachea and bronchopneumonia. |
| 52. | E. W. | 52 | F. | Squamous cell carcinoma left middle ear | R.T.<br>S. | 3<br>4 | I.A. | 80 | Single I.V. Doses; Systemic 40 mg./kg. Total of 17 g. | Full thickness skin loss. Marked oedema & blistering. Loss of pinna. Alopecia. Cystitis | Cavity clear of tumour for three months. Minimal healing | 2 for 7 months | I. A. chemotherapy combined with extensive surgery. Tumour has now extended anteriorly to temporo-mandibular joint. Patient comfortable. |
| 53. | M. W. | 69 | F. | Squamous cell carcinoma right middle ear (from meatus) | R.T.<br>S. | 4<br>6 | I.A. | 60 | Systemic Total of 18 g. | Minimal oedema and blistering with skin loss. Alopecia | Initial decrease in tumour | 2 for 4 months | I.A. chemotherapy combined with extensive surgery. Was never really well and lacked spirit. Postmortem—no macroscopic tumour. Bronchopneumonia. |
| 54. | A. B. | 61 | F. | Squamous cell carcinoma left antrum | R.T.<br>S. | 3<br>1 | | | I.A. 800 mg.<br>Systemic Total of 12 g. | Alopecia | Tumour sloughed from cavity | 1 for 2 months | Post mortem—residual tumour in orbit. Cerebral thrombosis. |
| 55. | G. L. | 48 | F. | Transitional cell and anaplastic carcinoma right antrum | R.T.<br>S. | 12<br>8 | I.A.<br>I.A. | 50<br>100 | Systemic Total of 10 g. | Severe oedema & blistering with skin loss. Alopecia | Initial improvement not maintained | 1 for 5 months | Post mortem—tumour extends to cranial cavity involving brain and pituitary. |

### Table I.—*continued.*

| | | | | Previous treatment | | Treatment | | | | | | | |
|---|---|---|---|---|---|---|---|---|---|---|---|---|---|
| | | | | | | Ethoglucid | | Cyclophosphamide | | | | | |
| Patient | Age | Sex | Site and nature of tumour | Method* | Months before chemotherapy | Route | Dosage (mg./kg.) | Route | Dosage | Complications | Objective response | Results† | Comments |
| 56. H. N. | 80 | M. | Transitional and squamous cell carcinoma R. antrum | R.T.<br>S. | 16<br>4 | I.A. | 50<br>100 | Systemic | Total of 2·4 g. | Severe leucopenia | Minimal tumour response | 0 for 1 month | Post mortem—acute tracheo - bronchitis. Tumour very small. |
| 57. L. A. | 14/12 | M. | Rhabdomyosarcoma right temporal bone | | | | | I.A.<br>Systemic | 12·8 mg./kg.<br>Total of 4 g. | | Tumour apparently controlled | 3 for 2 months | Died after developing signs of cerebral irritation. No post mortem. |
| 58. D. R. | 71 | M. | Squamous cell carcinoma subglottic larynx | R.T.<br>S. | 60<br>14 | | | Systemic | Total of 8·8 g. | | No response | 0 for 1 month | Recurrence in left bronchus and right second rib. No post mortem. |
| 59. O. R. | 64 | M. | Cylindroma and anaplastic carcinoma left parotid | R.T.<br>S. | 48<br>49 | I.A. | 45 | Systemic | 2 mg./kg./day 44 g. to date | | Decrease in swelling | 3 for 5 months | Recurrence in lateral wall of nasopharynx responded rapidly and patient remains well. |
| 60. G. R. | 50 | M. | Metastatic anaplastic carcinoma cervical nodes. Primary unknown | R.T.<br>S. | 24<br>6 | | | Systemic | 2 mg./kg./day 27 g. to date | Alopecia. Depression | Recurrence previously | 2 for 5 months | Cyclophosphamide given to control the undiscovered primary lesion and prevent further recurrences. |
| 61. J. T. | 61 | M. | Squamous carcinoma left pyriform fossa with cervical metastases | R.T.<br>S. | 12<br>1 | | | Systemic | Total of 18 g. | | | 1 for 2 months | Tumour at block dissection adherent to carotid. Post mortem—bronchopneumonia, pharyngeal fistula. Carcinoma infiltrating carotid. |
| 62. E. D. | 62 | F. | Squamous cell subglottic | S. | 8 | | | Systemic | Total of 18 g. | Nausea and vomiting | No response | 0 for 5 months | Recurrence in anterior wall of pharynx above tracheostomy. For radiotherapy after chemotherapy failure. |
| 63. J. R. | 79 | M. | Transitional cell supraglottic | | | | | Systemic | Total of 4·6 g. | | No response | 0 for 1 month | Post mortem—extensive growth, bronchopneumonia. |
| 64. L. L. | 53 | F. | Chondrosarcoma right temporal bone | R.T. | 5 | I.A. | 40 | Systemic | 3 mg./kg./day 13g. to date | Marked oedema & full thickness skin loss. Alopecia. Nausea | Tumour free cavity | 2 for 3 months | Tumour destroyed in past by Epodyl. In pain, but at home. |
| 65. M. S. | 51 | F. | Squamous carcinoma left middle ear | R.T.<br>S. | 24<br>30 | I.A. | 58 | Systemic | 3 mg./kg./day 9 /g. to date | Oedema, alopecia, nausea | No response | 0 for 3 months | The tumour picked up no I.A. dye and had enlarged with much pain. At home. |
| 66. R. B. | 45 | M. | Squamous carcinoma left tongue, cervical and skin metastases | R.T.<br>S. | 2<br>12 | | | Single I.V. doses<br>Systemic | 33–50 mg/kg.<br>3–6 mg./kg./day | Alopecia, cystitis, nausea, vomiting | Complete disappearance | 2 for 3 months | An enormous fungating skin recurrence disappeared on massive intravenous therapy with re-epithelialisation. At home. |
| 67. J. B. | 66 | M. | Squamous carcinoma left tonsillolingual sulcus | R.T.<br>S. | 8<br>4 | | | I.A.<br>Single IV doses | 40 mg./kg.<br>23–36 mg./kg. | Alopecia, nausea, vomiting | Partial improvement | 2 for 2 months | Pain relieved and ulcer diminished after I.A. injection. Progress not maintained. |
| 68. F. D. | 52 | M. | Squamous carcinoma supraglottic | S. | 3 | | | Systemic | Total of 17 g. | Nausea | No response | 0 for 2 months | Rapid metastatic spread post operatively & development of pulmonary tuberculosis. Post mortem—bony and myocardial metastases. |

| | | | | | | | | | | | | | |
|---|---|---|---|---|---|---|---|---|---|---|---|---|---|
| 69. S. S. | 63 | F. | Generalised lymphosarcoma | | | | | I.V. | Tota of 2 g. | | Tonsillar lesions responded dramatically | 1 | Died with parolytic ileus? due to tumours. Moribund on admission. No post mortem. |
| 70. W. F. | 77 | M. | Carcinoma-supraglottic | R.T. | 24 | | | Systemic | Total of 8 g. | | Swallowing improved | 1 for 1 month | Post mortem showed bronchopneumonia and myocardial degeneration. Tumour extensive. |
| 71. H. R. | 66 | M. | Squamous carcinoma supraglottic | R.T. S. | 48 24 | | | I.V. | 38 mg./kg. | | No response | 0 | Massive fistula. Discharged home at own request. |
| 72. P. B. | 40 | M. | Adenocarcinoma right antrum | R.T. S. | 36 35 | | | Single I.A. & I.V. doses | 40–47 mg./kg. | Alopecia, nausea, and vomiting | Proptosis disappeared | 2 for 2 months | May need further surgery. |
| 73. F. W. | 77 | M. | Poorly differentiated squamous carcinoma left pyriform fossa | | | | | I.A. Single I.V. doses | 40 mg./kg. 30 mg./kg. | Alopecia, nausea and vomiting | Virtually complete disappearance of tumour | 3 for 2 months | No previous therapy—tumour picked up dye poorly but surrounding mucosa intensely stained. |
| 74. G. B. | 7 | M. | Sarcoma post nasal space | R.T. | 1 | | | Single I.V. doses | 41–100 mg./kg. | Alopecia, nausea | Slight | 0 for 1 month | Post mortem—suppurative bronchopneumonia. Extensive tumour and metastatic necrosis. |
| 75. A. R. | 65 | F. | Squamous carcinoma left tonsil | R.T. S. | 7 5 | | | Single I.V. doses | 40 mg./kg. | Nausea and vomiting | Excellent | 2 for 2 months | Fungating tumour has sloughed. Biopsy of crater negative. |
| 76. R. T. | 55 | F. | Squamous carcinoma Supraglottic | R.T. S. | 4 18 | | | Systemic | 3 mg./kg./day 13 g. to date | Alopecia | | 0 for 2 months | Cervical swelling less. |
| 77. D. M. | 53 | F. | Squamous carcinoma subglottic | S. | 10 | | | Systemic | 3 mg./kg./day 15 g. to date | | | 0 for 2 months | Fistula remains ISQ. |
| 78. B. C. | 40 | F. | Squamous carcinoma of post cricoid | R.T. | 3 | | | Single I.V. doses | 44–50 mg./kg. | Alopecia, nausea, vomiting and diarrhoea | | 0 for 1 month | Dysphagia remains complete. |
| 79. J. F. | 32 | F. | Undifferentiated tumour nasal septum | R.T. S. | 24 2 | | | Single I.V. doses | 40 mg./kg. | | | 1 for 1 month | |
| 80. E. B. | 72 | F. | Anaplastic squamous carcinoma of nasopharynx & L. ethmoid | R.T. | 18 | I.A. | 150 | | | Slight pharyngeal oedema | Proptosis disappeared | 1 for 1 month | Tumour sloughed. |

### CHOICE OF CHEMOTHERAPEUTIC AGENT

At present two main groups of cytotoxic agents are available for the treatment of head and neck malignancies—the alkylating agents and the anti-metabolites. The former are generally toxic to all cells and are potent mutagenic agents. In many ways alkylating agents and ionizing radiations appear to produce similar biological effects.

However, different alkylating agents may vary in their effect upon a given tissue although these variations are possibly only quantitative, dependant upon the rate of alkylation with cell nucleic acids.

Anti-metabolites on the other hand act as competitive inhibitors of essential metabolic processes. Those most used in cancer chemotherapy are the anti-folic acid compounds and the purine and pyrimidine substances. The inhibition of the synthesis of nucleic acid would seem to be a logical attack against the neoplastic cell. Unfortunately published results indicate that an initially favourable response is rarely maintained, and the agent needs to be applied to the neoplastic cell for long periods in order to exert an effect upon fresh generations of dividing cells. Drug resistance invariably occurs, possibly due to the malignant cell utilising alternative pathways for the synthesis of essential metabolites.

In view of the disappointing results reported using the anti-metabolite methotrexate in head and neck cancer, it was decided to confine our therapy to the alkylating agents, ethoglucid and cyclophosphamide.

The best known alkylating agent is of course nitrogen mustard but the limiting factors in its use are its haematological toxicity, low therapeutic index and its non selective site of activity (Gilman and Philips, 1946). A report circulated in 1960 by A. L. Walpole (1960, personal communication) detailed the effect of a bis-epoxide, triethyleneglycol diglycidyl ether upon the Walker carcinoma 256 in rats. The experimental results were so striking in comparison with other alkylating agents that although appreciating the dangers of applying such results to human cancer, it was decided to use this compound for intra-arterial infusion in clinical neoplasms.

In many instances, however, local invasion or wide dissemination of a cancer makes regional intra-arterial infusion impracticable. For these cases cyclophosphamide was chosen, since it could be given by mouth and was inactive until the active radical is liberated at sites of high phosphatase and phosphamidase concentrations—as was thought to exist in tumour cells.

### A. ETHOGLUCID (TRIETHYLENEGLYCOL DIGLYCIDYL ETHER)

Inhibition of growth of the Walker tumour in rats by certain bis-epoxides given intra-peritoneally, was reported by Hendrey *et al.* in 1951. Development of this work showed that the bis-epoxide resorcinol diglycidyl when given intravenously had a marked inhibitory effect upon tumour growth, causing cytological changes of a radiometric type in both tumour and bone marrow. Walpole (personal communication) reported on the effect of triethyleneglycol diglycidyl ether on the Walker tumour in rats. Intravenous injection starting 24 hours after implantation caused complete suppression of tumour development in a significant proportion of the animals. Complete regression also occurred in tumours already established for five to six days—a unique occurrence.

*Physical and chemical properties*

In the pure state ethoglucid is a colourless liquid—specific gravity 1·13, miscible in all proportions with water and most organic solvents. It solidifies at low temperatures and melts between −15° C. and −11° C. Chemically it is highly reactive, giving in general addition products with acids and polymers with bases. Even when pure it appears to polymerise slowly and should be kept below 0° C.

Fig. 1.—Venous blood levels of ethoglucid following intravenous administration of 250 mg./kg

*Distribution and metabolism*

A method for the estimation of the compound in blood and tissues has been developed by Imperial Chemical Industries, Ltd., using *p*-hydroxyazobenzene *p*-sulphonic acid as a reagent. The rapid removal of ethoglucid from the blood is typical of alkylating agents and indicates that the drug is being localised in the tissues before being metabolically degraded. The results after intravenous (Fig. 1) and intra-arterial injection (Fig. 2) in man shows a metabolic half-life of 10–15 minutes. Ethoglucid has been shown to be present in large amounts in all the organs of the rat, except the liver, kidney, lung and ovary—after an intravenous injection of 300 mg./kg. There is free diffusion into the C.S.F. and human tumours can be seen to " wet " after intra-arterial injection.

Excretion of unchanged ethoglucid in the urine is negligible and tissue binding of the drug does not appear to be an important feature. Metabolic degradation is therefore, the only way of accounting for the rapid disappearance of the drug.

FIG. 2.—Venous blood levels of ethoglucid following intra-arterial administration of 250 mg./kg.

*Toxicity*

(a) *Local toxic effects*

Ethoglucid has only been given intra-arterially in this series of patients. Dosage is between 50 and 250 mg./kg. and the drug is diluted with normal saline. No incidence of vascular spasm or thrombosis has occurred. Leakage into surrounding tissues will produce vesiculation of the skin and necrosis.

(b) *Systemic toxic effects*

1. *Acute toxicity*.—Intra-arterial injection is always carried out under general anaesthesia. A moderate fall in blood pressure invariably occurs following injection and lasts approximately two minutes. Leakage into the soft tissues of the head and neck is accompanied by oedema and occasionally, blistering of the skin 6–8 hours after injection (Fig. 3). However in three patients the oedema was unusually severe and accompanied by profound hypotension and death. It is possible that the fatalities were the result of extreme histamine sensitivity and pre-operative skin testing with 1/10,000 histamine diphosphate is now carried out on all cases to be treated with this drug. In patients showing severe reaction the dosage injected is greatly reduced.

2. *Haematological effects.*—In man given a dose of between 150 and 250 mg./kg. the total white cell count will be depressed to its lowest value by the 14th day ($\pm$ 2 day) after injection. Values below 1000/c.mm. may be reached but recovery has been complete in every case by the 21st–35th day. Since reducing the dosage given intra-arterially to below 100 mg./kg. no total white cell count has fallen below 1000/c.mm. Polymorphs are most severely affected and may fall to below 10 per cent of the total count. No buccal ulceration has occurred in any patient even though total white counts of 500/c.mm. have been reached. The platelet count is also depressed reaching a minimum figure of under 30,000/c.mm. in severe cases. No serious bleeding has occurred and megakaryocytes are still found in the marrow. Some reduction in haemoglobin levels is common after repeated injections and blood transfusion may be necessary. Several patients have survived three intra-arterial injections repeated at four weekly intervals with no permanent damage to the haemopoietic system.

3. *Tumour necrosis.*—Unfortunately a dramatic tumour response may prove fatal to the patient. Rapid necrosis of the cancer may lead to inhalation bronchopneumonia, uncontrollable haemorrhage or the production of a large tracheo-oesophageal fistula. Where possible the intra-arterial injection is preceeded by the excision of as much neoplasm as possible combined with ligature of potentially dangerous blood vessels.

4. *Alopecia.*—Infusion of any alkylating agent into the branches of the superficial temporal artery will produce unilateral alopecia. Digital pressure is exerted over the main trunk of this vessel during the actual injection and minimises the risk of this unpleasant side effect.

### B. CYCLOPHOSPHAMIDE (N,N-BIS($\beta$-CHLOROETHYL)-N′,O-PROPYLENE PHOSPHORIC ACID DIAMIDE)

The N-phosphorylated nitrogen mustards, inactive precursors of the cytotoxic mustards, are thought to be activated by phosphoramidases and phosphatases. These enzymes are abundant in some malignant tumours and the development of cyclophosphamide by Arnold and Bourseaux in 1958 was an attempt to create an inactive transport form of an alkylating agent that would only become active within the tumour cell. The drug is supplied in tablet form for oral use, and as a powder to be dissolved in sterile water when intravenous or intra-arterial injection is indicated.

Cyclophosphamide is unique amongst alkylating agents since, being stable in aqueous solution and non-vesiculating, it can be given by almost any route. In this series of patients the drug has been administered intra-venously, intra-arterially, per orally and by direct injection into the tumour. Dosage has varied from a maintenance level of 4 mg./kg./day to single intravascular injections of 60 mg./kg.

### *Toxicity*

(a) *Local toxic effects*

Cyclophosphamide is very stable *in vitro.* No hydrolysis of the chlorine irons has been detected in a neutral aqueous solution during an experimental period of two hours (Brock, 1958). Injections are well tolerated and no signs of irritation of veins or arteries have been seen in patients of any age. The drug, when dissolved in normal saline, may be given intramuscularly, intra-peritoneally or intra-pleurally—all without producing discomfort.

(b) *Systemic toxic effects*

Cyclophosphamide has been given in this series of patients either as daily intravenous or oral doses, 4–8 mg./kg./day, or more recently, as single massive intravascular injections, 40–60 mg./kg. Systemic toxic effects will therefore be considered as resulting from daily dosage or single massive injections.

1. *Nausea and vomiting*

*Daily dosage* : 23/44 patients developed this unpleasant side effect at some stage of their treatment. There appears to be no direct correlation between the incidence of symptoms and either the level of daily dosage or total dosage. In most cases, giving the total daily dose at night together with Avomine controlled the nausea.

*Single massive injections* : 13/18 patients suffered severely from sickness soon after injection. However if they were not affected by the first injection then future injections with the same dosage did not produce nausea or vomiting.

2. *Haematological effects.*—The dosage of cyclophosphamide, given by any route, is limited by its most serious side effect—bone marrow depression. This is reflected, for practical purposes, in the peripheral blood picture. The total white cell count is estimated at least thrice weekly during the time the patient is in hospital. It is important to appreciate that the normal total white cell count in the peripheral blood varies from 4000–11,000/c.mm. of blood (Whitby and Britton, 1963). In addition the day to day variation in total white cell count in any patient is considerable and we have placed more importance on the upward and downward trend shown by successive counts than on any one individual count. The error in estimation of the total white cell count is of the order of $\pm$ 20 per cent. As the accepted lower limit of normality is 4000/c.mm. the daily dosage has been adjusted to produce a deliberate leucopenia of half this figure. The lower the daily dosage the longer the total white cell count takes to show a significant drop (Fig. 4) and in those patients where it proved impossible to maintain this leucopenia, there was no tumour response or alternatively " tumour escape ". There have been no cases of buccal ulceration despite prolonged eucopenia.

With single massive injections the time taken before a significant depression of the total white cell count occurs is independent of the dose injected. However the lowest count obtained *is* dependent on the total dosage given (Fig. 6) but varies with each individual patient. No helpful information has been obtained from differential counts and these have been discontinued.

---

EXPLANATION OF PLATE

FIG. 3.—Blistering of the facial skin following intra-arterial ethoglucid.

FIG. 5.—(*a*) Severe alopecia after prolonged administration of cyclophosphamide.

(*b*) Histological section of the scalp of a patient with alopecia showing thin dermis and absent bulbs of hair follicles.

FIG. 8.—Fungating neoplasm of the maxilla.

3

5a

8

5b

Harrison and Tucker.

16/44 patients had platelet counts carried out when the total white cell count was at its lowest level. 8/16 showed a level below 100,000/c.mm. but there has been no instance of spontaneous bleeding and these estimations are no longer carried out routinely. There is insufficient evidence to determine whether this drug has a selective effect upon the haemoglobin concentration but regular estimations are carried out and whole blood transfusions given when necessary.

3. *Alopecia.*—Noticeable loss of hair occurred in 22/44 patients receiving daily dosage and 6/8 on single massive injections. However the degree of alopecia is impossible to determine accurately but no obvious relationship was found bet-

FIG. 4.—Graph illustrating that the lower the daily dosage of cyclophosphamide the longer the total white cell count takes to be depressed to 2000/c.mm.

ween the onset of hair loss and total dose of cyclophosphamide or depression of total white cell count. Hair loss did appear to be greater in young women and the hair growing at the vertex apparently less resistant to epilation. Neither the eyebrows nor the lashes were involved (Fig. 5).

In the majority of cases surviving the early months of therapy, the hair regrew—in one patient with previously straight hair, the new growth was curly. Wigs are provided when requested.

4. *Cystitis.*—7/44 patients receiving a total of over 10 g. by daily dosage, developed a sterile cystitis. Eighteen patients on single massive injections had a total of forty doses but only one developed cystitis. However the remaining patients all had a high fluid intake for the twenty-four hours following the injection resulting in prolonged diuresis. The work of Mellett (1963) with tritium labelled cyclophosphamide (C-$^3$H) given to dogs at a dosage of 1·0 mg./kg. by intravenous injection, showed that 43 per cent of the total dose was excreted unchanged in the urine within twenty-four hours. The sterile cystitis that commonly occurs after prolonged or high dosage, may be an irritant effect of the high concentration of cyclophosphamide, or a breakdown product, in the bladder. The passage of large quantities of dilute urine secondary to high fluid intake appears to minimise this unpleasant side effect.

### METHODS OF ADMINISTRATION

Although the prognosis for patients with malignancies of the head and neck is poor there are certain advantages to be gained by treating them with chemotherapy.

FIG. 6.—Graph showing that the lowest total white cell count is dependent upon total dosage.

Firstly the survival time, even of " failed " cases, can be measured in several months and this gives the chemotherapeutic agent a reasonable opportunity of producing tumour regression. Secondly, in most cases the tumour mass can be easily seen and any change in size or appearance will be obvious. Biopsies can be taken at regular intervals without inconveniencing the patient and necrotic tumour tissue removed when necessary. Thirdly, use can be made of the fact that most head and neck tumours are supplied by branches of the external carotid artery. This vessel is readily available to the surgeon although previous radiotherapy or radical surgery may have destroyed much of the normal regional blood supply, thus reducing the efficacy of any intra-arterial chemotherapy.

Recent work by Goldacre and Sylvén (1962) on "the access of blood borne dyes to various tumour regions ", using experimental tumours, has substantiated our own experiences with Disulphine Blue (I.C.I.) in human neoplasms. Most of the early, small tumours appear to be well vascularised and stained intensely. However, the larger tumours frequently have a necrotic avascular area which does not usually stain. Biopsy shows that in this area there remain cells which histologically appear both malignant and viable—this has been confirmed experimentally by Goldacre and Sylvén (1962). Very occasionally these necrotic areas are seen to stain slightly but the colour remains for several days after the dye has disappeared from the rest of the body. We are in complete agreement with the comment of Goldacre and Sylvén, namely " the necrotic centre (of a tumour) is an uneven dispersion of living tumour cells surviving almost anaerobically in a medium of autolysed tumour tissue, which has no blood supply and exchanges material only very slowly with the external living tissue ". It is quite possible that the regrowth of tumour, which frequently occurs even after an initially satisfactory response to chemotherapy, may be initiated by active malignant cells lying in this avascular and untreated area.

It is our practice, whenever possible, to remove all necrotic tumour material before commencing chemotherapy. This not only reduces the mass of tissue to be treated, but after injection of Disulphine Blue ensures that all the visible tumour is stained and thus vascularised. Ethoglucid possesses the attribute of easy permeability and tumours may be seen to " wet " after intra-arterial infusion of this drug. This is particularly important with those tumours whose blood supply has been reduced because of previous treatment, or when avascular areas can not be excised.

Local tumour resection will reduce the dangers of toxic absorption from massive tumour necrosis and minimise the risk of inhalation of necrotic growth when the neoplasm is in close relation to the laryngeal inlet.

*Arterial supply to the head and neck*

The efficacy of any chemotherapeutic agent depends to some extent on whether it is possible to administer a large enough concentration to the tumour area. In most tumours this is dependent upon the vascular supply and has been discussed earlier in this paper. Most workers have used the main trunk of the external carotid as the afferent artery but in this series of cases we have not hesitated to infuse into both common and internal carotid arteries where necessary. No deleterious effects upon the brain have been observed with ethoglucid or cyclophosphamide.

In order to minimise leakage of the alkylating agent into the systemic circulation, it is desirable to administer the smallest effective dosage of the drug. It is therefore essential to isolate, where possible, the arterial supply to the tumour area. This can only be carried out by surgical exposure of the upper inch of the common carotid artery, the carotid bulb and the external carotid artery as high as possible. Any branches leaving these vessels must be identified, and those not required for infusion can be temporarily occluded. It is only by direct exposure that the many variations in the branching of the external carotid artery can be appreciated and care then taken to ensure that the cytotoxic agent is not infused into unwanted tissues.

(*a*) In 16 per cent of cases the superior thyroid arises from the common carotid artery. This vessel not only supplies the thyroid gland but gives off the superior laryngeal artery.

(*b*) The ascending pharyngeal, usually the smallest branch of the external carotid artery, arises in 14 per cent of cases from the occipital artery and not from the posterior surface of the external carotid. It supplies the pharynx, branches to the palate and tonsil and the inferior tympanic branch to the middle ear.

(*c*) In 20 per cent of cases the lingual and facial arteries arise together from a common stem. This is extremely important as the facial artery is much larger and if both vessels are not identified then the greater proportion of any drug introduced into the common trunk will pass into the face and not the tongue! Occasionally the facial artery arises in common with the internal maxillary artery.

There is of course considerable overlapping of the vascular supply to most regions of the head and neck and the problem is further complicated by previous radiotherapy or surgery. After identification of the branches of the external carotid artery, methylene blue is injected into the main vessel. This ensures that :—

(1) the blood supply to the tumour has been identified—verified by staining of the tumour, and

(2) there is no staining of unwanted tissues. The latter is not always avoidable where the vessel supplying the tumour gives off branches which cannot be occluded. Disulphine Blue is then injected—this is also a tracer dye but by binding with plasma proteins produces a more intense and permanent colour. This is of value for photography and post-operative inspection of the tumour—it is excreted unchanged by the kidneys within 36 hours.

As yet no attempt has been made to expose the thyro-cervical trunk although this will be essential if complete control of the vascular supply to larynx, thyroid gland and musculature of the neck is to be gained.

*Intra-arterial chemotherapy*

There has been an embarrassing amount of literature relating to both the technique and complications of indwelling arterial catheters (Nahum, 1962). Arterial cannulation is essential with the antimetabolites but with the short acting alkylating agents a much simpler technique has been employed, reducing the demands upon both nursing staff and hospital accommodation.

A. *Ethoglucid.*—The pharmacology of this bis-epoxide has been discussed previously, sufficient to say that it is an active alkylating agent and has been given intra-arterially in 30 patients in this series.

After confirmation that the blood supply to the tumour area has been isolated ethoglucid, diluted with normal saline, is slowly infused through the same hypodermic needle used previously to inject the tracer dye. Dilution of the cytotoxic agent is necessary to prevent irritation and spasm of the vessel wall and varies from four to ten times depending upon the size of the artery. If the needle has been placed close to the carotid bulb, then 1 per cent procaine is introduced before the ethoglucid to minimise the risk of a carotid sinus reflex.

At the completion of the infusion, the needle is removed and pressure applied to the artery. The neck incision is then closed. During the administration of the ethoglucid there is usually a temporary fall in blood pressure and if the tumour is well vascularised, its surface may be seen to " wet " indicating that the drug has permeated the whole tumour mass.

In the earlier cases the ethoglucid was given in doses of between 200 and 250 mg./kg. This has now been reduced to under 100 mg./kg. with equal tumour effect but marked reduction in toxic side effects. If necessary this dose may be repeated, when any depression of the total white cell count has recovered. Within twenty-four hours the main tumour mass will slough and this may be accompanied by haemorrhage, toxic absorption or even inhalation of necrotic debris. The latter particular applies to lesions of the laryngo-pharynx. Some oedema of surrounding tissues is invariable and follows leakage of the drug away from the tumour or through non-occluded branches of the afferent vessel. Intravenous Phenergan 25 mg is now given immediately pre-operatively and appears to prevent the severe, and often lethal oedema which occurred in some earlier cases.

When tumours of the tongue or laryngo-pharynx are being treated a preliminary tracheotomy is carried out as a precaution against severe laryngeal oedema. If possible these patients are now treated with cyclophosphamide especially if intra-dermal injection of 1/10,000 histamine diphosphate produces a marked histamine sensitivity response. Samples are taken from the venous drainage of the tumour (either the common facial or internal jugular vein) at regular intervals during the infusion. This necessitates cannulating the vein and the concentration of ethoglucid is estimated utilising a relatively simple technique (Fig. 2).

B. *Cyclophosphamide.*—Ten patients have been given this drug intra-arterially, the afferent vessel to the tumour area being isolated using the same technique as described for ethoglucid. Initially a standard dosage of 600 mg. dissolved in 300 ml. of normal saline was infused using a simple gravity feed. More recently the dosage has been increased to 40 mg./kg. Infusion is now carried out by means of a pump and the solution warmed to 40° C. to increase tumour blood flow. Nahum and Rochlin (1963) have suggested body cooling to achieve a differential temperature gradient between the tumour and systemic tissues, together with a vasodilator such as papaverine hydrochloride in the warmed infusate. This would appear to be a logical approach to this problem and it is intended to modify our technique in future cases. Sloughing of the tumour mass occurs within twenty-four hours but is not accompanied by oedema of surrounding tissues.

*Systemic Chemotherapy*

Unfortunately in many patients previous treatment will have radically affected the vascular anatomy of the head and neck making intra-arterial chemotherapy impracticable. Systemic metastases or widespread local extension may also indicate that the most effective treatment is by the intravenous or oral route.

(a) *Low dosage maintenance therapy.*—In this regime, employed in earlier cases but now replaced by single massive intravenous therapy, cyclophosphamide was given in daily intravenous injections of 8 mg./kg./day, until the total white cell count had fallen to 2000/c.mm. The drug was then given by mouth in sufficient quantity—usually 4 mg./kg./day to maintain this leucopenia. The patient was seen at regular intervals for white cell counts and appraisal of tumour response, and treatment continued in successful cases for at least a year or until tumour "escape" occurred. Nausea was a frequent complication but could be controlled by anti-emetics or by taking the daily dose at night together with a mild hypnotic.

(b) *High dose therapy.*—Single intravenous injections of 40–60 mg./kg. are given and repeated when the leucopenia which follows has recovered (Fig. 7). This large dose produces severe nausea and vomiting for twelve hours and patients are admitted to hospital for the day. Fluid intake is increased for several days and this avoids the unpleasant chemical cystitis which invariably occurs secondary to the excretion of a high concentration of cyclophosphamide. Clinical results utilising this technique have been most rewarding especially in cases previously untreated.

FIG. 7.—Effect of repeated single massive intravenous injections of cyclophosphamide on the total white cell count. Case 66.

### RESULTS OF TREATMENT

Any attempt to select patients for chemotherapy on purely ethical grounds is in our opinion, not only impossible but unrealistic. In this series no patient has been refused treatment, no matter how extensive the primary lesion or how widespread the metastases.

A positive biopsy was obtained in every case (Table I), 84 per cent being squamous carcinomata. It is our impression that the degree of differentiation of these tumours plays little part in determining the tumour response to ethoglucid or cyclophosphamide. Many investigators have attempted to assess tumour response to chemotherapy by devious, intricate and imaginative techniques. The majority of tumours encountered in this series have been extensive and the patients so desperate that our prime aim has been to produce at the very least, symptomatic relief, and at best a return to normal life. Results tabulated in

Table I have been graded on clinical and sociological findings and not on macroscopical and radiological impressions.

*Grade 0—No effect*

In 26 patients (32 per cent) no worthwhile improvement was produced—this included all cases dying within one month of commencing treatment. Most of these patients were elderly and either had extensive disease with metastases or had previously received treatment by radical surgery and radiotherapy. No case with a local recurrence of growth after radical dissection of glands of the neck showed any improvement with systemic chemotherapy.

*Grade 1—Symptomatic relief*

Twenty-four patients (30 per cent) were recorded as showing symptomatic relief. This grading defies accurate definition but includes any case showing for at least two months, reduction in tumour size, cessation of pain or the return of some previously impaired function. In 3 cases the rate of growth of the tumour was controlled for over six months and it was only on the cessation of treatment that rapid growth of the neoplasm resulted in death of the patient. No case was able to return home for more than a few days.

*Grade 2—Home with limited activity*

In 21 cases (26 per cent) improvement was enough to enable the patient to return home. Limited medical and nursing care was still necessary in most cases but the patient was encouraged, and able, to live as active a life as the individual circumstances allowed. Every case eventually returned to hospital and the period of home-care varied from two to eighteen months. To some extent these patients became once again, the responsibility of the family doctor although regular visits to hospital for blood counts and tumour appraisal were insisted upon whenever feasible. We consider this group of patients to have been the most rewarding. All were incurable by present day techniques and the most that chemotherapy could offer was to reduce the size of the tumour and relieve pain or toxicity long enough to enable the patients to return to their home without dependence upon relatives or friends. With very extensive tumours in unfavourable sites such as the nasopharynx, middle ear and ethmoidal labyrinth, this is probably the greatest improvement that will be possible in the foreseeable future.

*Grade 3—Return to normal life*

The 9 patients (12 per cent) placed in this group all showed disappearance of their tumours whilst under treatment. Two have died from other causes and histological examination of post mortem material revealed no residual malignancy. All of the remaining patients are now at home living completely normal lives. Case 5 and Case 6 first started chemotherapy twenty-two months ago and Case 7 twenty months ago. Re-evaluation of this small group of patients has revealed no obvious explanation for their exceptional good response to treatment.

### CAUSES OF DEATH

Every patient accepted for chemotherapy has been re-admitted if requested during the terminal stage of their disease. Consequently considerable experience has been gained not only of deaths directly attributable to chemotherapy but also of the natural history of advanced cancer of the head and neck.

(a) *Age*

Sixty per cent of the patients treated were aged 60 years or over, although ages varied from 14 months to 82 years. Many were extremely ill and feeble as a result of malnutrition, toxaemia or concurrent cardiovascular and respiratory disease. Treatment was never refused on the grounds of age alone although several successful tumour responses were followed by gradual deterioration and death a few weeks later.

(b) *Extension of the tumour*

Even a rapid increase in the rate of growth of tumours of the head and neck rarely results in immediate death unless a major blood vessel is involved or dura exposed with subsequent meningitis. Tumours, especially of the maxilla, may grow to considerable proportions causing the unfortunate patient great distress but without producing death for many months (Fig. 8). The passage of feeding and tracheotomy tubes provides the patient with food and air but may only prolong an already unhappy existence.

(c) *Destruction of tumour*

Inhalation bronchopneumonia frequently accompanies both natural and chemotherapeutic destruction of large tumours of the laryngo-pharynx. Where the tumour has replaced a party wall, as exists between trachea and cervical oesophagus, then destruction of the growth may result in a large dehiscence incompatable with life. Two patients died as a direct result of infection through such a defect although subsequent post mortem examination showed almost complete tumour destruction.

(d) *Overwhelming infection*

We have recently been concerned over the apparent lack of resistance to infection shown by a few patients receiving long term systemic cyclophosphamide. After a total dosage exceeding 10 g. three patients succumbed to overwhelming chest infections. Leucopenia is unlikely to have been the underlying factor as this is deliberately produced and maintained in most cases. It is our impression that these patients had lost their power of immune body response, perhaps secondary to cyclophosphamide induced atrophy of the reticulo-endothelial system.

With the exception of two cases, every patient in this series dying in hospital has had a post mortem examination carried out. This has provided valuable information as to the effect of chemotherapy on head and neck tumours—paths of extension of uncontrolled neoplasms and the incidence of unsuspected systemic metastases. The data obtained from this investigation, based on 42 post mortem examinations, will be incorporated into a future paper.

### DISCUSSION

There is considerable confusion as to the rightful role that chemotherapy should play in the management of cancer of the head and neck. Chemotherapeutic success depends largely on concentrating enough of the active agent within the tumour area—but without producing fatal systemic side effects. Although avascular necrotic tumour tissue can be excised, previous radical surgery or radiotherapy destroys much of the regional blood supply making it impossible to bring enough active cytotoxic agent into close contact with the neoplastic cell. However it would be difficult at present, to justify the thesis that all cancers of the head and neck should be treated primarily by chemotherapy.

The majority of patients reviewed in this paper had already failed to respond to orthodox treatment and faced an often prolonged period of misery and disability before finally succumbing to haemorrhage or bronchopneumonia. It is our contention that the practical value of a chemotherapeutic agent can only be determined by its use in cases of human cancer. The changing from one preparation to another, without a properly conducted clinical trial, is to be strongly deprecated. Only recently have we felt reasonably assured of our ability to effectively administer the two alkylating agents described in this paper.

Eight patients, with previously untreated neoplasms have received chemotherapy as their primary therapy. In each case the prognosis was poor because of advanced age, systemic disease etc. However the immediate response has been dramatic and it is intended to use this form of therapy in further selected cases. The potentialities of effective chemotherapy are legion but it is wise to remember that as yet the available cytotoxic agents are only of limited value in head and neck cancer. Our experience has shown that both ethoglucid and cyclophosphamide, when administered selectively, can offer considerable relief to patients with advanced cancer of the head and neck, providing vascular supply remains. Lymph gland metastases, particularly in a neck previously irradiated or operated upon, presents an almost insuperable problem—as does the very advanced neoplasm.

If the extremely poor prognosis of malignant conditions of the head and neck is to be improved then our attention must be turned towards the diagnosis and management of the early lesion. When surgical excision is appropriate this could be combined with single intravenous injections of cyclophosphamide 40 mg./kg. ; it is fully appreciated however that the rationale of this proposal could not be substantiated or refuted for many years. An attempt might also be made to prevent dissemination of tumour cells by intra-arterial injection of ethoglucid before surgical excision. Our own search for circulating cancer cells in the regional veins has proved unsuccessful in this series of cases. Both these proposals are now implemented in our management of new cases of head and neck cancer.

Enthusiastic attempts to improve the prognosis and well-being of these unfortunate patients is praiseworthy but may be unjustifiable when resulting in severe side effects or suppurating deformities. The selection of both drugs and techniques described in this paper was carried out with these principles in mind and the result only termed successful when the patient returned home, able to live a normal existence.

### SUMMARY

Eighty cases of advanced cancer of the head and neck have been treated with intra-arterial or systemic chemotherapy over the past two years. Two alkylating agents, ethoglucid and cyclophosphamide have been used and their pharmacology and methods of administration is discussed in detail. Nine patients

(12 per cent) obtained complete regression of their tumours, three remaining alive and well at the present time—22 months after commencing chemotherapy. Some of the factors which may be important in influencing the efficacy of chemotherapy are examined together with suggestions for improving the prognosis in early neoplasms of the head and neck.

Acknowledgements must be made to the Consulting and Nursing Staff of the Royal National Throat, Nose and Ear Hospital, London, for the provision and care of these patients : to Professor I. Friedmann and Dr. D. A. Osborn of the Institute of Laryngology and Otology for bearing the ever increasing burden of haematological and histological investigations. We are indebted to Mr. D. J. Connolly and staff of the Department of Clinical Photography for their patience and care in preparing the illustrations for this paper.

We should like to express our thanks to the many colleagues both in this country and abroad, who have referred patients to us for treatment and to both Imperial Chemical Industries Ltd., (Pharmaceutical Division), and Ward Blenkinsop & Co. Ltd., without whose assistance this work would not be possible. One of us (W.N.T.) is a Duveen Research Fellow of the University of London.

### REFERENCES

Arnold, H. and Bourseaux, F.—(1958) *Naturwissenschaften*, **45**, 64.
Austin, W. G., Monaco, A. P., Richardson, G. S., Baker, W. H., Shaw, R. S. and Baker, J. W.—(1959) *New Engl. J. Med.*, **261**, 1037.
Bierman, H. R., Kelley, K. H., Byron, R. L., Dod, K. S. and Shimkin, M. B.—(1951) *J. nat. Cancer Inst.*, **11**, 891.
Brock, N.—(1958) *Arzneimitt. Forsch.*, **8**, 1.
Cooling, C. I., Garai, O. and Staunton, M. D.—(1962) *Brit. med. J.*, i, 1231.
Creech, O.—(1958) *Ann. Surg.*, **148**, 616.
*Idem*, Krementz, E. T., Ryan, R. F., Reemtsma, K. and Elliott, J. L.—(1959) *J. Amer. med. Ass.*, **171**, 2069.
Gilman, A. and Philips, F. S.—(1946) *Science*, **103**, 409.
Goldacre, R. J. and Sylvén, B.—(1962) *Brit. J. Cancer*, **16**, 306.
Goodman, L. S., Wintrobe, M., Dameshak, W., Goodman, M. J., Gilman, A. and McLennan, M. T.—(1946) *J. Amer. med. Ass.* **132**, 126
Hendry, J. A., Homer, R. F., Rose, F. L. and Walpole, A. L.—(1951) *Brit. J. Pharmacol.*, **6**, 235.
Hickey, R. C., Johnson, C. A., Evans, T. C. and Alftine, D.—(1959) *Arch. Surg., Chicago*, **79**, 416.
Jacobson, L. O., Spurr, C. L., Barron, E. S., Guzman, S. T., Lusbrough, C. and Dick, G. F.—(1946) *J. Amer. med. Ass.*, **132**, 263.
Klopp, C. T., Alford, T. C., Bateman, J., Berry, G. N. and Winship, T.—(1950) *Ann. Surg.* **132**, 811.
Knock, F. E.—(1959) *Surg. Gynec. Obstet.*, **109**, 445.
Krementz, E. T., Creech, O., Ryan, R. F., Reemtsma, K. and Winblad, J. N.—(1960) *Acta. Un. int. Cancr*, **26**, 874.
*Iidem* and Elliott, J. L.—(1959) *Proc. Amer. Ass. Cancer Res.*, **3**, 34.
Mellett, L. B.—(1963) *Fed. Proc.*, **22**, 305.
Milnes Walker, R., Espiner, H. J. and Vowles, K. D. J.—(1962) *Lancet*, i, 177.
Nahum, A. M.—(1962) *Surg. Gynec. Obstet.*, **115**, 478.
*Idem* and Rochlin, D. B.—(1963) *Amer. J. Surg.*, **105**, 759.
Pierpont, M. and Blades, B.—(1960) *J. thoracic cardiov. Surg.*, **39**, 159.

Reemtsma, K. A., Ryan, R. F., Krementz, T. and Creech, O.—(1959) *Arch. Surg., Chicago*, **78**, 724.
Rhoads, C. P.—(1946) *J. Amer. med. Ass.*, **131**, 656.
Shingleton, W. W., Reeves, J. W. Jnr., Keppel, R. A., Mamaley, S. and Taylor, H. M.—(1959) *Ann. Surg.*, **151**, 741.
Stehlin, J. A., Clark, R. L., White, E. L., Smith, J. L., Griffin, A. C., Jesse, R. M. and Healey, J. E.—(1960) *Ibid.*, **151**, 605.
Sullivan, R. D., Jones, R., Schnabel, T. G. and Shorey, J. M.—(1953) *Cancer*, **6**, 121.
*Idem*, Miller, E. and Sikes, M. P.—(1959) *Ibid.*, **12**, 1248.
Westbury, G.—(1963) *Ann. Roy. Coll. Surg. Engl.*, **32**, 358.
Whitby, L. E. H. and Britton, C. J. C.—(1963) ‘ Disorders of the blood ’ 9th Edition, London (Churchill).
Woodhall, B., Hall, K., Mamaley, S. and Jackson, J.—(1959) *Ann. Surg.*, **150**, 690.